# A novel hydrogen persulfide donor HPD1 alleviated LPS-induced neuroinflammation by modulating glial polarization through TLR4-MAPK/NF-κB pathway

**DOI:** 10.1016/j.redox.2026.104430

**Published:** 2026-10-08

**Authors:** Fei Yan, Shouwei Tao, Yan Jiao, Ting Kang, Shuangqing Du, Yue Cheng, Zhenbo Huang, Ping Feng, Bowen Ke

**Affiliations:** a Clinical Trial Center, National Medical Products Administration Key Laboratory for Clinical Research and Evaluation of Innovative Drugs, West China Hospital, Sichuan University, Chengdu, Sichuan, 610041, China; b Department of Anesthesiology, Laboratory of Anesthesia and Critical Care Medicine, National-Local Joint Engineering Research Centre of Translational Medicine of Anesthesiology, West China Hospital, Sichuan University, Chengdu, Sichuan, 610041, China; c Key Laboratory of Tropical Biological Resources of Ministry of Education, School of Pharmaceutical Sciences, Hainan University, Haikou, Hainan, 570228, China

**Keywords:** Neuroinflammation, Neuroprotection, Phenotypic polarization, Hydrogen persulfide, Hydrogen sulfide, TLR4-MAPK/NF-κB signaling

## Abstract

The biological role of hydrogen persulfide (H_2_S_2_) has remained elusive due to the scarcity of controllable donors, despite its importance as a reactive sulfur species whose dyshomeostasis drives neuroinflammation. Herein, we employed HPD1, a novel esterase-responsive H_2_S_2_ donor, to investigate its anti-inflammatory effects and underlying mechanisms in lipopolysaccharide (LPS)-stimulated glial cells. HPD1 exhibited superior cytoprotective and anti-inflammatory efficacy over inorganic sodium disulfide (Na_2_S_2_) and sodium hydrosulfide (NaHS) in BV2 microglia, consistent with its superior and sustained intracellular H_2_S_2_ release efficiency. Functionally, HPD1 dose-dependently attenuated LPS-induced cytotoxicity and shifted microglia and primary astrocytes from pro-inflammatory, neurotoxic states toward anti-inflammatory, neuroprotective phenotypes, as characterized by M1-to-M2 and A1-to-A2 marker profiles, respectively. Mechanistically, HPD1 downregulated toll-like receptor 4 (TLR4) and attenuated mitogen-activated protein kinase (MAPK) activation, as evidenced by reduced extracellular signal-regulated kinase 1 and 2 (ERK1/2), p38, and c-Jun N-terminal kinase (JNK) phosphorylation in BV2 microglia. In both BV2 microglia and primary astrocytes, HPD1 markedly suppressed the phosphorylation of both the p65 subunit of nuclear factor-κB (NF-κB) and inhibitor of κBα (IκBα), prevented the proteasomal degradation of IκBα, and blocked the nuclear translocation of p65. Consequently, HPD1 attenuated the release of neuroinflammatory mediators while promoting the production of neuroprotective factors, thereby protecting neuronal HT22 cells against neurotoxicity induced by conditioned medium from LPS-activated BV2 microglia. In vivo, HPD1 reduced serum proinflammatory cytokine levels in both an acute intrathecal LPS-induced neuroinflammation model and a chronic constriction injury-induced neuropathic pain model. Collectively, our findings identify the TLR4-MAPK/NF-κB axis as a potential molecular pathway through which sustained H_2_S_2_ delivery by HPD1 reprograms glial phenotypic polarization and promotes neuroprotection. This study provides mechanistic insights into H_2_S_2_ signaling and supports the therapeutic potential of controllable H_2_S_2_ supplementation for neuroinflammatory disorders.

## Highlights

• A novel esterase-responsive H_2_S_2_ donor HPD1 exhibits superior cytoprotective and anti-inflammatory effects over inorganic H_2_S and H_2_S_2_ donors.
• HPD1 dual-regulates microglial M1/M2 and astrocytic A1/A2 polarizations to alleviate neuroinflammation, thereby preserving neuronal damage.
• The anti-inflammatory mechanism of HPD1 relies on blocking the TLR4-MAPK/NF-κB signaling cascade.
• H_2_S_2_ supplementation represents a promising therapeutic strategy for neuroinflammation-related diseases.

## Graphical abstract

**Figure:**
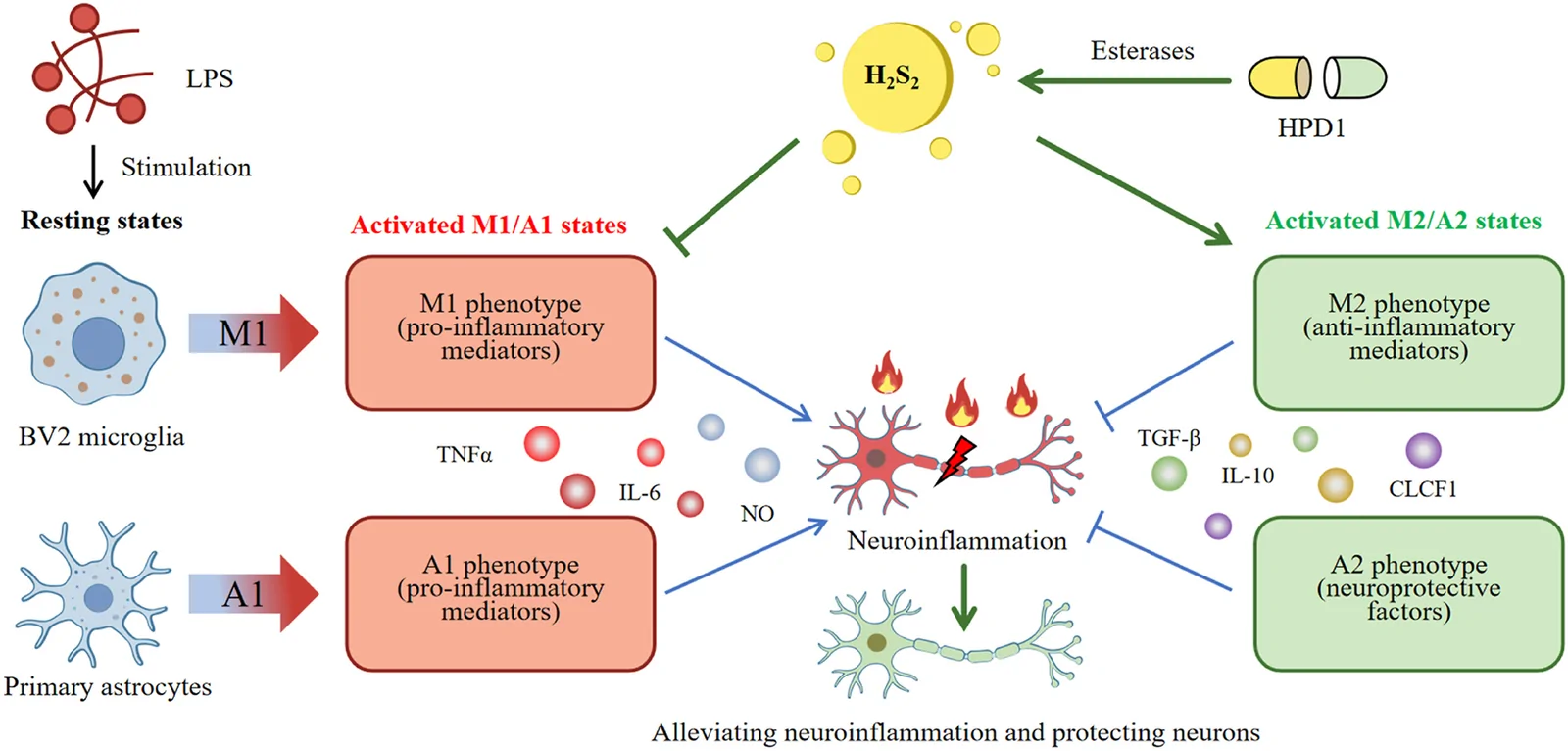

## 1 Introduction

The dysregulation of reactive sulfur species (RSS) homeostasis is intimately linked to glial-mediated neuroinflammation, which is critically implicated in the pathogenesis of various central nervous system (CNS) disorders, including neurodegenerative diseases, chronic pain, stroke, and traumatic brain injury [1,2]. Accumulating evidence has revealed a significant decline in endogenous RSS levels across these pathological conditions, and this deficiency acts as a pivotal driver of neuroinflammation by triggering the activation of glial cells [[3], [4], [5]].

Reprogramming glial phenotypic polarization and suppressing pro-inflammatory pathways have remained the core therapeutic strategy to mitigate neuroinflammation. In response to sustained pathological insults, microglia and astrocytes are activated and consequently exhibit an imbalance in phenotypic polarization between the pro-inflammatory M1/A1 phenotypes and the neuroprotective M2/A2 phenotypes, respectively [[6], [7], [8]]. In experimental models, lipopolysaccharide (LPS)-induced neuroinflammation is widely characterized by the imbalanced phenotypic polarization of glial cells. LPS-induced M1 microglia polarization is characterized by the robust expression of neurotoxic inflammatory mediators, ranging from pro-inflammatory receptors such as toll-like receptor 4 (TLR4) and surface markers like cluster of differentiation 86 (CD86), to enzymes including inducible nitric oxide synthase (iNOS) and cyclooxygenase-2 (COX-2), as well as critical cytokines and chemokines such as interleukin-1β (IL-1β), tumor necrosis factor-α (TNFα), interleukin-6 (IL-6), and CXC motif chemokine ligand 10 (CXCL10) [7]. Conversely, neuroprotective M2-polarized microglia are typically suppressed, a state marked by the diminished expression of anti-inflammatory mediators such as transforming growth factor-β (TGFβ), macrophage mannose receptor (CD206), interleukin-10 (IL-10), and arginase-1 (ARG1) [9]. Similar to microglia, astrocytes undergo A1 polarization in vitro upon exposure to either a specific cytokine triad (interleukin-1α, TNFα, and component 1q) or conditioned medium from activated microglia [6,10]. Likewise, LPS treatment directly drives the transformation of A1-like reactive astrocytes [11,12], evidenced by specific A1-type markers such as complement component 3 (C3) and C1 inhibitor (C1-INH), thereby exerting neurotoxic effects mediated by the production of pro-inflammatory mediators. Concurrently, the neuroprotective A2-like phenotype is compromised, as characterized by the downregulation of its markers including S100 calcium-binding protein A10 (S100A10), cluster of differentiation 109 (CD109), and cardiotrophin-like cytokine factor 1 (CLCF1) [6]. The imbalanced glial polarization thus serves as a primary driver of neuroinflammation, and suppressing pro-inflammatory pathways and restoring the M1/M2 and A1/A2 balances have emerged as leading therapeutic strategies for neuroinflammatory diseases. Accumulating research suggests that the imbalance in glial polarization is governed by multiple signaling pathways [13,14], most notably the canonical TLR4-mediated signaling pathway. Upon stimulation by LPS, TLR4 is activated to trigger the downstream mitogen-activated protein kinase (MAPK) and nuclear factor-κB (NF-κB) signaling cascades, thereby initiating the expression of pro-inflammatory mediators to drive M1/A1 phenotypes while suppressing M2/A2 phenotypes, ultimately amplifying inflammatory and neurotoxic effects [6,11,15]. Importantly, numerous studies have reported that modulating the TLR4-MAPK/NF-κB pathway can effectively restore this phenotypic balance [[16], [17], [18]], offering a viable strategy to mitigate neuroinflammation. Specifically, hydrogen sulfide (H_2_S) supplementation has been investigated to modulate microglial and astrocytic polarization [19,20], thereby mitigating neuroinflammation; however, its pharmaceutical efficacy remains suboptimal.

H_2_S_2_ supplementation is emerging as a more significant pharmacological strategy than H_2_S due to its greater potency and unique biological profile. H_2_S_2_ and H_2_S are key members of the RSS family that extensively regulate inflammatory responses and redox balance in the CNS [21,22]. Notably, it has been reported that the concentration of H_2_S_2_ in the mouse brain is 26 nmol/g protein, comparable to that of H_2_S (30 nmol/g protein) [23]. However, research on the physiological and pharmacological effects of H_2_S_2_ remains more limited than that on H_2_S, largely due to its inherent instability and high reactivity. While H_2_S_2_ is considered to share overlapping physiological effects with H_2_S, accumulating evidence highlights fundamental distinctions between the two RSS. The superior nucleophilicity and reducing capacity of H_2_S_2_, stemming from its lower pKa value relative to H_2_S (5.0 *vs* 7.0) [24], facilitate the highly efficient scavenging of free radicals, protecting key proteins from cellular and tissue damage induced by oxidative stress [25]. H_2_S_2_ functions as a critical endogenous metabolite and signaling molecule of H_2_S [26,27], rather than merely serving as its “sulfur pool” [28]. Distinct from H_2_S due to its electrophilicity, H_2_S_2_ enables direct protein thiol persulfidation without intermediate oxidation steps [29,30], which directly drives sulfur-mediated signaling pathways, effectively conferring a protective mechanism against oxidative-inflammatory stress [25,31]. Moreover, we previously demonstrated that the H_2_S_2_ donor displays greater antinociceptive potency and fewer side effects than the H_2_S donor [32]. Altogether, prioritizing pharmacological research on H_2_S_2_ may yield more significant therapeutic insights.

Nevertheless, the development of H_2_S_2_ donors remains scarce, and in-depth mechanistic insights into H_2_S_2_ actions are still limited. In this study, we therefore employed HPD1, a novel esterase-responsive H_2_S_2_ donor designed and synthesized in our laboratory [33], to compare its therapeutic profile with inorganic donors (sodium hydrosulfide, NaHS, a H_2_S donor; and sodium disulfide, Na_2_S_2_, a H_2_S_2_ donor) and elucidate its underlying mechanisms in LPS-stimulated glial cells. We demonstrated that HPD1 displayed superior cytoprotective and anti-inflammatory efficacy over Na_2_S_2_, whereas NaHS exerted negligible effects, which closely paralleled its higher intracellular H_2_S_2_ release efficiency. Furthermore, HPD1 conferred neuroprotection by regulating glial phenotypic polarization and rebalancing M1/M2 microglial and A1/A2-like astrocytic phenotypes in LPS-stimulated BV2 microglia and primary astrocytes, an effect strongly correlated with the inhibition of the canonical TLR4-MAPK/NF-κB signaling pathway. For in vivo study, HPD1 attenuated serum proinflammatory cytokine levels in acute intrathecal LPS-induced neuroinflammation and chronic constriction injury (CCI)-induced neuropathic pain models, underscoring its therapeutic applicability to inflammatory disorders.

## 2 Materials and methods

### 2.1 Cell cultures and drug treatment

#### 2.1.1 BV2 and HT22 cell lines

The murine microglial BV2 and neuronal HT22 cells were cultured as described previously [34,35]. Briefly, cells were cultured in high-glucose Dulbecco's modified Eagle's medium (DMEM; #C11995500BT, Gibco, New York, NY, USA) supplemented with 10% fetal bovine serum (FBS; #F0193, Sigma-Aldrich, St.Louis, MO, USA), 100 U/mL penicillin and 100 μg/mL streptomycin (#PYG0016, Boster, Wuhan, China) in humidified incubator at 37 °C with a 5% CO_2_ atmosphere. The culture medium was refreshed every 2 to 3 days. Cells were dissociated with 0.25% Trypsin-EDTA (#25200056, Gibco) and passaged at a 1:4 split ratio once they achieved 80%-90% confluency.

#### 2.1.2 Primary astrocyte

Primary astrocytes were isolated from the cerebral cortices of postnatal day 1 Institute of Cancer Research (ICR) mice as described previously [36]. Under a dissecting microscope, the cerebral cortices were harvested and meticulously stripped of the meninges and blood vessels in ice-cold D-Hanks (#PYG0079, Boster), and all residual blood clots were carefully cleared prior to enzymatic digestion. The tissues were dissociated in 0.25% trypsin (#25200056, Gibco) for 7 min at 37 °C, followed by mechanical trituration. The resulting cell suspension was passed through a 70 μm cell strainer (#BS-70-CS, Biosharp, Hefei, China) to remove tissue debris. The mixed glial cells were seeded into T75 flasks pre-coated with poly-D-lysine (100 μg/mL; #ST508, Beyotime, Shanghai, China) and cultured in DMEM (#C11995500BT, Gibco) supplemented with 10% FBS (#35-081-CV, Corning Inc., Corning, NY, USA), 100 U/mL penicillin and 100 μg/mL streptomycin (#PYG0016, Boster), which were kept at 37 °C in a humidified atmosphere with 5% CO_2_. After 10 days, the flasks were shaken at 200 rpm at 37 °C for 2 h, and the floating cells were discarded. The remaining microglia and oligodendrocytes were removed beforehand via digestion with 0.25% trypsin for 5-7 min. Subsequently, a second 10 to 15-min digestion was performed on the remaining astrocytes, which were then collected and maintained in 10% FBS-containing medium to establish a baseline reactive state rather than a resting state [10], under which subsequent LPS challenge induced a pro-inflammatory “A1-like” reactive phenotype. Immunocytochemical analysis confirmed a culture purity of > 95%, as defined by glial fibrillary acidic protein (GFAP)-positive cells.

#### 2.1.3 In vitro drug treatments

Following the culture methods described above, BV2 microglial cells (4 × 10^4^ cells/cm^2^) or primary astrocytes (1 × 10^5^ cells/cm^2^) were seeded in 96-well or 6-well plates and cultured overnight or for 48 h, respectively. For comparative efficacy assays, BV2 cells were co-treated with LPS (1 μg/mL; #L8880, Solarbio, Beijing, China) and 100 μM of either HPD1, NaHS (#S106641-25g, Aladdin Biochemical Technology Co., Ltd., Shanghai, China), or Na_2_S_2_ (#SB02, Dojindo Molecular Technologies, Inc., Kumamoto, Japan) for 24 h. For dose-response experiments, BV2 microglia or primary astrocytes were treated with varying concentrations of HPD1 (2, 10, 50, and 100 μM) in the presence or absence of LPS (1 μg/mL; #L8880, Solarbio) for 24 h. Following the incubations, the culture supernatants were collected from the 6-well plates, and the cells were harvested or processed for the indicated downstream experiments.

### 2.2 Animals and drug administration

The experimental protocols involving animals were approved by the Animal Research Committee of West China Hospital of Sichuan University (approval No. 20220311021), complying with the NIH Guide for the Care and Use of Laboratory Animals and the ethical guidelines of the International Association for the Study of Pain. Male ICR mice (6-8 weeks old; Chengdu Dossy Experimental Animals Co., Ltd., Chengdu, China) were housed in pathogen-free facility under controlled temperature (23 ± 1 °C) and a 12-h light/dark cycle (lights on 07:00), with food and water ad libitum.

LPS-induced acute neuroinflammation model: Acute spinal neuroinflammation was induced via intrathecal injection of LPS as previously described [37]. Under isoflurane anesthesia, the caudal back hair was depilated, and the animal was immobilized pronely by stabilizing the pelvis. The L5-L6 intervertebral space was located via palpation, into which a 30G needle attached to a 10-μL Hamilton Gastight syringe (#20976, Sigma-Aldrich) was introduced transcutaneously through the midline. Successful spinal access was verified by the sudden elicitation of a brisk tail-flick response.

CCI-induced neuropathic pain model: Peripheral neuropathic pain was induced by CCI of the sciatic nerve as described previously [38]. Under isoflurane anesthesia, the left sciatic nerve was surgically exposed at the mid-thigh level via blunt dissection. Three chromic gut sutures (4-0) were tied loosely around the nerve proximal to its trifurcation with 1-mm space intervals, ensuring nerve constriction without arresting epineural blood flow. Sham-operated animals underwent an identical surgical procedure to expose the left sciatic nerve without nerve ligation.

Drug administration and serum collection: HPD1 was dissolved in a vehicle consisting of 10% dimethyl sulfoxide, 10% polyethylene glycol 400, and 80% sterile saline. For the acute LPS model, HPD1 (either 5 or 10 μg) was mixed with LPS (1 μg; #L8880, Solarbio) and co-administered intrathecally in a total volume of 5 μL. To prevent solution backflow and facilitate diffusion, the needle was maintained in place for an additional 15 s prior to withdrawal. Blood samples were collected 2 h after a single intrathecal injection. For the chronic CCI model, mice received a single intraperitoneal injection of HPD1 (14 mg/kg) or vehicle on post-operative day 7, and blood samples were collected 2 h post-injection. All harvested blood samples were allowed to clot, and the separated serum was collected for ELISA to determine the levels of inflammatory cytokines.

### 2.3 BV2 microglia-conditioned medium treatment of HT22 cells

To investigate the neuroprotective potential of HPD1 against microglia-induced neuronal injury, we established a cell damage model by treating HT22 cells with conditioned medium (CM) derived from BV2 microglia, as previously reported [35]. Briefly, BV2 microglial cells were seeded at a density of 5 × 10^5^ cells/well in 6-well plates, incubated overnight, and then co-treated for 24 h with HPD1 (10, 50, and 100 μM) and LPS (1 μg/mL; #L8880, Solarbio). The supernatants were collected and centrifuged at 1000 rpm for 5 min to remove dead cells and debris, yielding the CM. This CM was then used to completely replace the medium of HT22 neuronal cells pre-seeded at a density of 5 × 10^4^ cells/cm^2^ in 24- or 96-well plates. Following a 24-h incubation, HT22 cells were assessed for morphological changes, cell viability, and apoptosis.

### 2.4 MTT assay

Cell viability was assessed using the 3-(4,5-dimethylthiazol-2-yl)-2,5-diphenyltetrazolium bromide (MTT) assay [39]. Briefly, BV2 microglia, HT22 neuronal cells, or primary astrocytes seeded in 96-well plates were treated as indicated. The culture media were carefully aspirated and replaced with fresh culture medium containing 0.5 mg/mL MTT (#BS186-250 mg, Biosharp). Following a 1 h incubation at 37 °C, the supernatants were carefully removed, and 200 μL of dimethyl sulfoxide was added to each well to dissolve the formazan crystals. Plates were then shaken at room temperature for 10 min and the absorbance at 490 nm was measured by using a microplate reader (SpectraMax i3x, Molecular Devices, Sunnyvale, CA, USA). Cell viability was expressed as a percentage of the untreated control group.

### 2.5 Calcein-AM/PI assay

To evaluate the direct cell viability of BV2 microglia co-treated with LPS (1 μg/mL; #L8880, Solarbio) and 100 μM of either HPD1, NaHS, or Na_2_S_2_ as well as HT22 neuronal cells exposed to BV2-derived CM, a Calcein-AM/PI staining assay was conducted using a commercial kit (#C2015S, Beyotime) according to the manufacturer's instructions. Briefly, the culture medium was aspirated, and cells were washed once with phosphate buffered saline (PBS; #PYG0021, Boster). Subsequently, 250 μL of Calcein-AM/PI working solution was added to each well of the 24-well plate, followed by a 30-min incubation at 37 °C in the dark. After another PBS wash, the cells were visualized under an inverted fluorescence microscope (OLYMPUS IX83, Olympus Co., Tokyo, Japan), and live cells (green fluorescence) and dead cells (red fluorescence) were quantified using Fiji/ImageJ software.

### 2.6 Enzyme-linked immunosorbent assay

The levels of inflammatory cytokines (IL-6, IL-1β, and TNFα) in the culture supernatants of BV2 cells and primary astrocytes (harvested from 6-well plates) as well as in mouse serum samples were detected by QuikCyto mouse IL-6 (#EMC004QT.96, NeoBioscience, Shenzhen, China), IL-1β (#EMC001bQT.96, NeoBioscience), and TNFα (#EMC102aQT.96, NeoBioscience) ELISA kits, respectively, according to the manufacturer's guidelines. Finally, the absorbance value at 450 nm was then measured by a microplate reader (SpectraMax i3x, Molecular Devices).

### 2.7 NO content assay

Nitrite, a stable precursor of nitric oxide (NO), was quantified using a NO detection kit (#S0021S, Beyotime) following the manufacturer's instructions. Fifty microliters of the culture supernatant was mixed with 50 μL of Griess Reagent I and then with 50 μL of Griess Reagent II. The absorbance was measured at 540 nm using a microplate reader (SpectraMax i3x, Molecular Devices). Nitrite concentrations were calculated by referencing the standard curve of sodium nitrite.

### 2.8 RNA isolation and real-time quantitative PCR assay

Total RNA was extracted from BV2 cells and primary astrocytes using a FastPure Cell/Tissue Total RNA Isolation Kit V2 (#RC112-01, Vazyme, Nanjing, China), and 0.8 μg total RNA was used to reverse-transcribed into cDNA using the MetaScript qPCR RT SuperMix with dsDNase kit (#A504-02, EXONGEN, Chengdu, China) according to the manufacturer's instructions. Subsequently, mRNA expression levels were quantified via real-time quantitative PCR (RT-qPCR) assay employing UltraStart Universal SYBR qPCR Master Mix (#A411-01, EXONGEN). The reactions were performed on a Real-Time PCR System (CFX Connect, Bio-Rad, Hercules, CA, USA) under the following conditions: 95 °C for 3 min (1 cycle) followed by 40 cycles of 95 °C for 10 s and 57 °C for 30 s. The primer sequences (Tsingke, Beijing, China) used in this study are provided in Table 1. The expression data were normalized relative to the mRNA levels of *Gapdh* (encoding glyceraldehyde-3-phosphate dehydrogenase, GAPDH) and the results were evaluated using the 2^−ΔΔCt^ method.

**Table 1:** The primer sequences.

Genes (proteins)	Forward	Reverse	Product length
*Arg1* (ARG1)	CTCCAAGCCAAAGTCCTTAGAG	AGGAGCTGTCATTAGGGACATC	185
*C3* (C3)	AAAGCCCAACACCAGCTACA	GAATGCCCCAAGTTCTTCGC	115
*Cd109* (CD109)	GGGGAAATGGGCTCCCTATG	CATCACAATGGCGCTTCTGG	141
*Cd86* (CD86)	CTGGACTCTACGACTTCACAATG	AGTTGGCGATCACTGACAGTT	131
*Clcf1* (CLCF1)	CTTCAATCCTCCTCGACTGG	TACGTCGGAGTTCAGCTGTG	176
*Cxcl10* (CXCL10)	CCAAGTGCTGCCGTCATTTTC	GGCTCGCAGGGATGATTTCAA	157
*Gapdh* (GAPDH)	AAGGTCGGAGTCAACGGATTT	AGATGATGACCCTTTTGGCTC	352
*Il6* (IL-6)	TAGTCCTTCCTACCCCAATTTCC	TTGGTCCTTAGCCACTCCTTC	76
*Il10* (IL-10)	CTTACTGACTGGCATGAGGATCA	GCAGCTCTAGGAGCATGTGG	101
*Il1b* (IL-1β)	GCAACTGTTCCTGAACTCAACT	ATCTTTTGGGGTCCGTCAACT	89
*Mrc1* (CD206)	CTCTGTTCAGCTATTGGACGC	CGGAATTTCTGGGATTCAGCTTC	132
*Nos2* (iNOS)	GGAGTGACGGCAAACATGACT	TCGATGCACAACTGGGTGAAC	127
*Ptgs2* (COX-2)	TGCACTATGGTTACAAAAGCTGG	TCAGGAAGCTCCTTATTTCCCTT	124
*S100a10* (S100A10)	CTAGCCTCATCGTGGTGTGC	GAGTCTGTCGAAACCTGGGC	71
*Serping1* (C1-INH)	ACAGCCCCCTCTGAATTCTT	GGATGCTCTCCAAGTTGCTC	299
*Tgfb* (TGFβ)	TCGACATGGATCAGTTTATGCG	CCCTGGTACTGTTGTAGATGGA	147
*Tnf* (TNFα)	CAGGCGGTGCCTATGTCTC	CGATCACCCCGAAGTTCAGTAG	89

### 2.9 H_2_S_2_ release assay

To assess intracellular H_2_S_2_ release mediated by different sulfur donors (HPD1, NaHS, or Na_2_S_2_), the specific DSP-3 probe was employed to detect H_2_S_2_ levels in BV2 microglia [40]. BV2 cells seeded in 6-well plates were cultured overnight, washed twice with PBS buffer, and incubated with 10 μM DSP-3 probe in PBS for 30 min. After removing the excess probe by washing with PBS, the cells were treated in PBS for 40 min with: (1) 100 μM of either HPD1, NaHS, or Na_2_S_2_; or (2) varying concentrations of HPD1 (10, 50, and 100 μM). Following PBS washes, fluorescence signals were visualized using the Olympus IX83 inverted fluorescence microscope and quantified using Fiji/ImageJ software.

### 2.10 Flow cytometry

Cell apoptosis was evaluated using an Annexin V-FITC Apoptosis Detection Kit (#C1062 M, Beyotime) following the manufacturer's protocol. Briefly, after a 24-h treatment with BV2-derived CM, the culture supernatants were collected, and the remaining adherent HT22 cells were harvested via trypsinization. The cells and supernatants were then pooled and centrifuged at 1000 rpm for 5 min. The resulting cell pellets were resuspended in 200 μL of 1×binding buffer containing 5 μL of Annexin V-FITC and 10 μL of propidium iodide (PI) staining solution. After a 15-min incubation at room temperature in the dark, samples were placed on ice and analyzed within 1 h using a CytoFLEX flow cytometer (Beckman Coulter, Brea, CA, USA), with data processing performed using FlowJo (version 10.8.1) software.

### 2.11 Immunofluorescence

Immunofluorescence staining was conducted as previously reported [41]. Cells seeded in 29 mm glass-bottom confocal dishes (#D29-14-1.5-N, Cellvis, Mountain View, CA, USA) were fixed in 4% paraformaldehyde (#BL539A, Biosharp) for 30 min at room temperature, followed by three 5-min washes in PBS. Cells were then permeabilized with 0.2% Triton X-100 (#T8200, Solarbio) in PBS for 30 min and blocked with 10% normal goat serum (#C0265, Beyotime) for 1 h. Subsequently, the cells were incubated overnight at 4 °C with a rabbit anti-NF-κB p65 primary antibody (1:200; #10745-1-AP, Proteintech, Wuhan, China), washed with PBS, and then incubated with a CoraLite594-conjugated goat anti-rabbit secondary antibody (1:200; #SA00013-4, Proteintech) for 1 h at room temperature. After washing with PBS, cell nuclei were stained with DAPI (#0100-20, SouthernBiotech, Birmingham, AL, USA), and visualized using confocal microscopy (LSM 980 Airy Scan II, 63×, Zeiss, Oberkochen, Germany).

### 2.12 Western blotting

Western blotting was conducted as previously described to quantify protein expression levels [42]. Briefly, total proteins from BV2 cells and primary astrocytes were lysed using RIPA lysis buffer (#P0013B, Beyotime) supplemented with protease inhibitors (#HY-K0010, MCE, Monmouth Junction, NJ, USA) and phosphatase inhibitors (#abs9162, Absin, Shanghai, China). The cell lysates were incubated on ice for 30 min and then sonicated on ice using a probe ultrasonicator (VCX130, Sonics & Materials Inc., Newton, CT, USA) at 20 kHz for 20 s. After centrifugation at 12,000 rpm for 15 min at 4 °C, the supernatants were collected. Protein concentrations were determined using a BCA protein assay kit (#P0010, Beyotime) according to the manufacturer's instructions. Subsequently, protein denaturation was achieved by diluting the lysates in 5×loading buffer (#P0015L, Beyotime), followed by heating at 95 °C for 7 min. Equal amounts of protein were loaded and separated in 1×Tris-glycine running buffer (192 mM glycine, 25 mM Tris base, 0.1% SDS) using an Omni-Easy™ One-step Color PAGE Gel Rapid Preparation Kit (#PG212, Epizyme, Shanghai, China) at 150 V for 1 h at room temperature, and then transferred to polyvinylidene fluoride membranes (#IPVH00010, Millipore, Bedford, MA, USA) using the Trans-Blot® Turbo™ Transfer System (#1704150, Bio-Rad) at 1.4 A for 8 min. The membranes were blocked in 10% non-fat dry milk powder in TBST (#G0004-1L, Servicebio, Wuhan, China) for 2 h at room temperature, followed by overnight incubation at 4 °C with the primary antibodies (listed in Table 2). After washing with TBST, the membranes were incubated with the secondary antibodies (Table 2) for 1 h at room temperature. Protein bands were visualized using enhanced electrochemiluminescence reagent (#4AW011-100, 4A Biotech, Suzhou, China) and captured with the ChemiDoc XRS + Gel Imaging System (ChemiDoc XRS+, Bio-Rad). Band intensities were analyzed using the Image Lab (version 6.1.0, Bio-Rad), and the relative expression levels of target proteins were normalized to α-Tubulin or β-actin to avoid signal overlap.

**Table 2:** Antibodies used for Western blotting.

Antibody type	Target	Host	Reaction	Dilution	Manufacturer (Catalog)
Primary	α-Tubulin	Mouse	Mouse	1:5000	Proteintech (66031-1-Ig)
Primary	β-actin	Mouse	Mouse	1:5000	Proteintech (66009-1-Ig)
Primary	C3	Rabbit	Mouse	1:1000	Abcam (ab200999)
Primary	COX-2	Rabbit	Mouse	1:2000	Proteintech (12375-1-AP)
Primary	ERK1/2	Rabbit	Mouse	1:5000	Proteintech (11257-1-AP)
Primary	IκBα	Mouse	Mouse	1:5000	Proteintech (66418-1-Ig)
Primary	IL-6	Rabbit	Mouse	1:1000	CST (12912S)
Primary	iNOS	Rabbit	Mouse	1:2000	ABclonal (A3774)
Primary	JNK	Mouse	Mouse	1:4000	Proteintech (66210-1-Ig)
Primary	p38	Mouse	Mouse	1:3000	Proteintech (66234-1-Ig)
Primary	p65	Rabbit	Mouse	1:2000	Abcam (ab16502)
Primary	p-ERK1/2	Rabbit	Mouse	1:2000	Proteintech (28733-1-AP)
Primary	p-IκBα	Rabbit	Mouse	1:2000	Proteintech (82349-1-RR)
Primary	p-JNK	Rabbit	Mouse	1:2000	Proteintech (80024-1-RR)
Primary	p-p38	Rabbit	Mouse	1:2000	Proteintech (28796-1-AP)
Primary	p-p65	Rabbit	Mouse	1:5000	ABclonal (AP1294)
Primary	Pro-IL-1β	Goat	Mouse	0.25 μg/mL	R&D System (AF-401-NA)
Primary	S100A10	Rabbit	Mouse	1:1000	CST (70573T)
Primary	TLR4	Mouse	Mouse	1:4000	Proteintech (66350-1-Ig)
Primary	TNFα	Rabbit	Mouse	1:1000	CST (11948S)
Secondary	HRP-conjugated IgG (H + L)	Rabbit	Goat	1:5000	Boster (BA1060)
Secondary	HRP-conjugated IgG (H + L)	Goat	Mouse	1:10000	Proteintech (SA00001-1)
Secondary	HRP-conjugated IgG (H + L)	Goat	Rabbit	1:10000	Proteintech (SA00001-2)

### 2.13 Statistical analysis

The experimental data are presented as mean ± SEM of at least three independent experiments. Statistical significance was analyzed by one-way ANOVA followed by Tukey's multiple comparisons test using Graph Pad Prism 10.1.2 software (GraphPad Software Inc., Boston, MA, USA). *P* values of < 0.05 (two tailed) were considered statistically significant.

## 3 Results

### 3.1 HPD1 exhibited superior cytoprotective and anti-inflammatory effects over NaHS and Na_2_S_2_ in LPS-stimulated BV2 microglia

To evaluate the biological profile of our newly developed H_2_S_2_ donor HPD1, LPS-stimulated BV2 microglia were treated with HPD1 (100 μM), with NaHS (100 μM, an inorganic H_2_S donor) or Na_2_S_2_ (100 μM, an inorganic H_2_S_2_ donor) serving as reference controls. The chemical structure of HPD1 is shown in Fig. 1A. We first confirmed intracellular H_2_S_2_ release from HPD1 using the fluorescent probe DSP-3 (Fig. 1B) [40]. HPD1 markedly enhanced green fluorescence intensity and, at an equimolar concentration, produced a significantly stronger signal than Na_2_S_2_, demonstrating superior intracellular H_2_S_2_ release efficiency; in contrast, NaHS failed to produce elevated signals (Fig. 1C; HPD1 *vs* Control, *p* < 0.0001; Na_2_S_2_ *vs* Control, *p* = 0.0119; NaHS *vs* Control, *p* = 0.9281). Consistent with this intracellular H_2_S_2_ profile, Calcein-AM/PI live/dead staining (Fig. 1D) showed that HPD1 dramatically suppressed LPS-induced cell death, reducing both the dead cell count (Fig. 1E; *F*_(4, 10)_ = 77.20, *p* < 0.0001) and percentage (Fig. 1F; *F*_(4, 10)_ = 76.69, *p* < 0.0001) to near-baseline levels, an effect superior to that of Na_2_S_2_, while NaHS showed almost no efficacy. In agreement, the MTT assay further demonstrated that HPD1 completely restored cell viability in LPS-stimulated BV2 microglia and significantly outperformed Na_2_S_2_, whereas NaHS exerted negligible cytoprotective effects (Fig. 1G; *F*_(4, 15)_ = 24.33, *p* < 0.0001). Moreover, LPS induced a sharp increase in NO release, a key pro-inflammatory mediator; HPD1 markedly suppressed NO production by 83%, whereas Na_2_S_2_ achieved only 34% inhibition and NaHS exerted no significant inhibitory effect (Fig. 1H; *F*_(4, 15)_ = 958.8, *p* < 0.0001). Overall, these findings demonstrated that HPD1 provides robust cytoprotection and anti-inflammatory action in LPS-stimulated BV2 microglia, far outperforming the inorganic H_2_S and H_2_S_2_ controls.

**Fig. 1:**
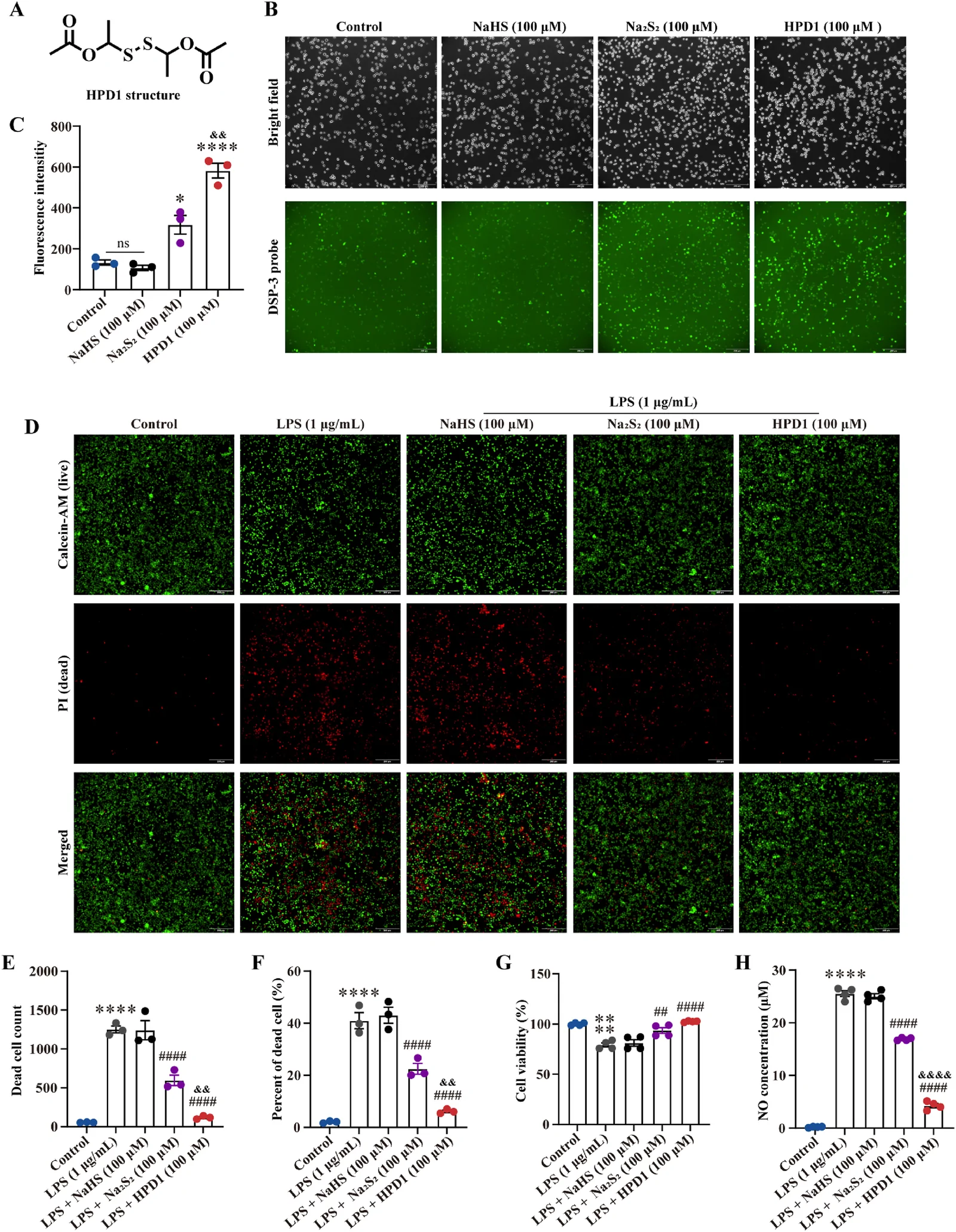
**HPD1 superiorly protected BV2 microglia against LPS-induced cell damage compared to NaHS and Na_2_S_2_.** **(A)** Chemical structure of HPD1. **(B, C)** Intracellular H_2_S_2_ levels detected by DSP-3 fluorescent probe: **(B)** representative brightfield and fluorescence images, and **(C)** quantitative analysis of fluorescence intensity in BV2 microglia pre-incubated with DSP-3 probe (10 μM) followed by treatment with NaHS (100 μM), Na_2_S_2_ (100 μM), or HPD1 (100 μM), scale bar = 200 μm (n = 3). **(D-F)** Representative fluorescence images **(D)** and quantitative analysis of dead cell count **(E)** and percentage of dead cells **(F)** in BV2 microglia stained with Calcein-AM (green, live cells) and PI (red, dead cells), scale bar = 200 μm (n = 3). **(G)** BV2 cell viability measured by MTT assay after treatment with LPS (1 μg/mL) in the presence or absence of NaHS, Na_2_S_2_, or HPD1 (n = 4). **(H)** NO concentration in cell culture supernatants measured by Griess assay (n = 4). Data are presented as mean ± SEM and analyzed using one-way ANOVA followed by Tukey's multiple comparisons test. *p < 0.05, ****p < 0.0001 *vs.* Control group; ##*p* < 0.01, ####*p* < 0.0001 *vs.* LPS-only group; &&*p* < 0.01, &&&&*p* < 0.0001 *vs.* Na_2_S_2_ group or LPS + Na_2_S_2_ group; ns, not significant. H_2_S_2_: hydrogen persulfide; LPS: lipopolysaccharide; MTT: 3-(4,5-dimethylthiazol-2-yl)-2,5-diphenyltetrazolium bromide; NaHS: sodium hydrosulfide; Na_2_S_2_: sodium disulfide; NO: nitric oxide; PI: propidium iodide.

### 3.2 HPD1 alleviated LPS-induced glial toxicity in parallel with intracellular H_2_S_2_ elevation

To further assess the cytoprotective and anti-inflammatory potential of HPD1, we examined its protective effects across a range of concentrations against LPS-induced damage in BV2 microglia and primary astrocytes. Primary astrocytes were isolated from neonatal ICR mouse cerebral cortices (Fig. 2A), and GFAP immunostaining confirmed a culture purity of 95% (Fig. 2B). The MTT assay demonstrated that HPD1 treatment alone exerted no significant cytotoxicity on BV2 microglia at concentrations up to 100 μM (Fig. 2C; *F*_(4, 20)_ = 0.1483, *p* = 0.9616). Notably, HPD1 treatment dose-dependently rescued the reduction in cell viability induced by LPS in both BV2 microglia (Fig. 2D; *F*_(5, 24)_ = 19.30, *p* < 0.0001) and primary astrocytes (Fig. 2E; *F*_(5, 24)_ = 21.94, *p* < 0.0001). Consistent with the viability profiles, morphological analysis revealed that the LPS-induced activated state of BV2 cells, characterized by an amoeboid-like phenotype (as indicated by the arrows), was substantially attenuated by HPD1, with cells reverting toward a resting morphology (Fig. 2F). Additionally, to determine whether the cytoprotective effects of HPD1 match intracellular H_2_S_2_ levels, we measured HPD1-mediated H_2_S_2_ release in BV2 microglia using the DSP-3 probe (Fig. 2G). In alignment with the protective dose-response profile, treatment with HPD1 at 10 μM failed to produce detectable signal elevation, whereas HPD1 treatment at 50 μM and 100 μM concentration-dependently induced a marked increase in intracellular H_2_S_2_ levels (Fig. 2H; HPD1 100 μM *vs* Control, *p* < 0.0001; HPD1 50 μM *vs* Control, *p* = 0.0101; HPD1 10 μM *vs* Control, *p* = 0.9737; HPD1 100 μM *vs* HPD1 50 μM, *p* = 0.0021). Taken together, these findings demonstrated that HPD1 effectively mitigates LPS-induced injury in both microglia and astrocytes in a dose-dependent manner, an effect intrinsically linked to its intracellular H_2_S_2_ release.

**Fig. 2:**
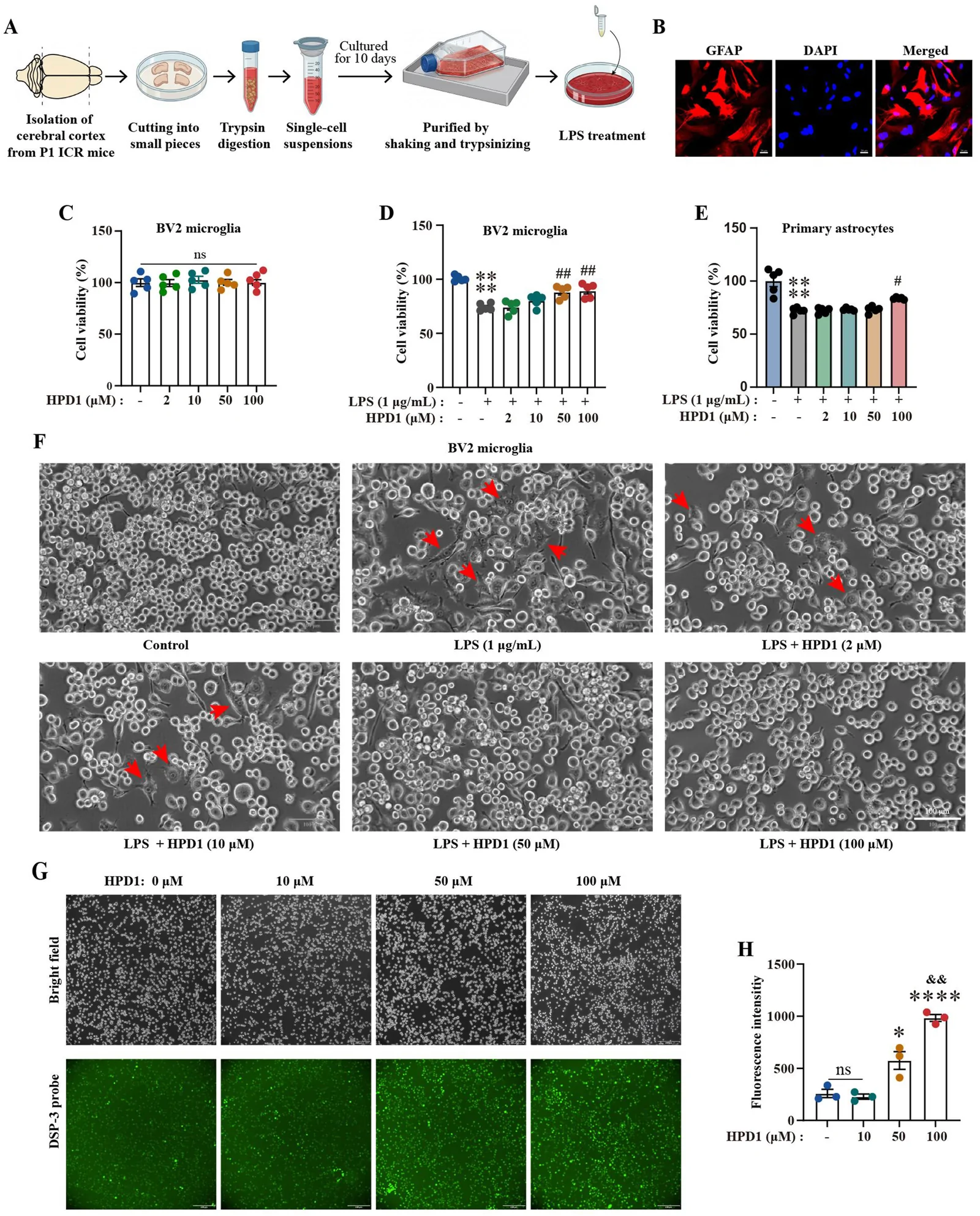
**HPD1 mitigated LPS-induced cell damage in BV2 microglia and primary astrocytes.** **(A)** Schematic diagram of the isolation and culture of primary astrocytes. **(B)** Representative immunofluorescence images of purity verification of primary astrocytes, GFAP, red; DAPI, blue; scale bar = 20 μm. **(C-E)** Cell viability determined by MTT assay in **(C)** BV2 microglia treated with indicated concentrations of HPD1 alone, and **(D)** BV2 microglia and **(E)** primary astrocytes co-treated with LPS (1 μg/mL) and indicated concentrations of HPD1 (n = 5). **(F)** Representative morphological images of BV2 microglia. Arrows indicated the activated amoeboid-like phenotype induced by LPS, which was reversed by HPD1 treatment, scale bar = 100 μm. **(G, H)** Intracellular H_2_S_2_ levels detected by the fluorescent probe DSP-3: **(G)** representative brightfield and fluorescence images, and **(H)** quantitative analysis of fluorescence intensity in BV2 microglia pre-incubated with DSP-3 probe (10 μM) followed by treatment with indicated concentrations of HPD1, scale bar = 200 μm (n = 3). Data are presented as mean ± SEM and analyzed using one-way ANOVA followed by Tukey's multiple comparisons test. *p < 0.05, ****p < 0.0001 *vs.* Control group; #*p* < 0.05, ##*p* < 0.01 *vs.* LPS-only group; &&*p* < 0.01 *vs.* HPD1 (50 μM) group; ns, not significant. GFAP: glial fibrillary acidic protein; H_2_S_2_: hydrogen persulfide; LPS: lipopolysaccharide.

### 3.3 HPD1 suppressed microglial M1 polarization

To investigate whether HPD1 can suppress neuroinflammation, we first evaluated its regulatory effect on the M1 pro-inflammatory phenotype in BV2 microglia. RT-qPCR results showed that LPS stimulation significantly upregulated the mRNA expression of M1 pro-inflammatory markers, including *Il6* (encoding IL-6, upregulated 4150.3-fold), *Il1b* (IL-1β, 1651.7-fold), *Tnf* (TNFα, 43.1-fold), *Ptgs2* (COX-2, 122.5-fold), *Nos2* (iNOS, 82.4-fold), *Cd86* (CD86, 1.9-fold), and *Cxcl10* (CXCL10, 76.3-fold) in BV2 cells. HPD1 treatment dose-dependently suppressed the mRNA levels of these genes, reaching a lowest 33% (*Cd86*) to highest 84% (*Ptgs2*) reduction at 100 μM relative to the LPS-only group (Fig. 3A–G). Consistently, Western blot analysis confirmed that HPD1 treatment dose-dependently attenuated the LPS-induced upregulation of key inflammatory mediators. LPS stimulation markedly increased the protein expression of IL-6, pro-IL-1β, TNFα, COX-2, and iNOS by 3.1- to 64.4-fold, whereas treatment with 100 μM HPD1 suppressed these protein levels by 2.2-fold (IL-6) to 4.2-fold (TNFα) compared to the LPS-only group (Fig. 3H–M). Furthermore, ELISA and Griess assays demonstrated that 100 μM HPD1 treatment significantly blocked the release of inflammatory mediators into the cell culture supernatants, suppressing IL-6, TNFα, and NO levels by 95%, 90%, and 84%, respectively (Fig. 3N–P). Collectively, these findings indicated that HPD1 effectively inhibits microglial M1 polarization and exerts potent anti-inflammatory effects.

**Fig. 3:**
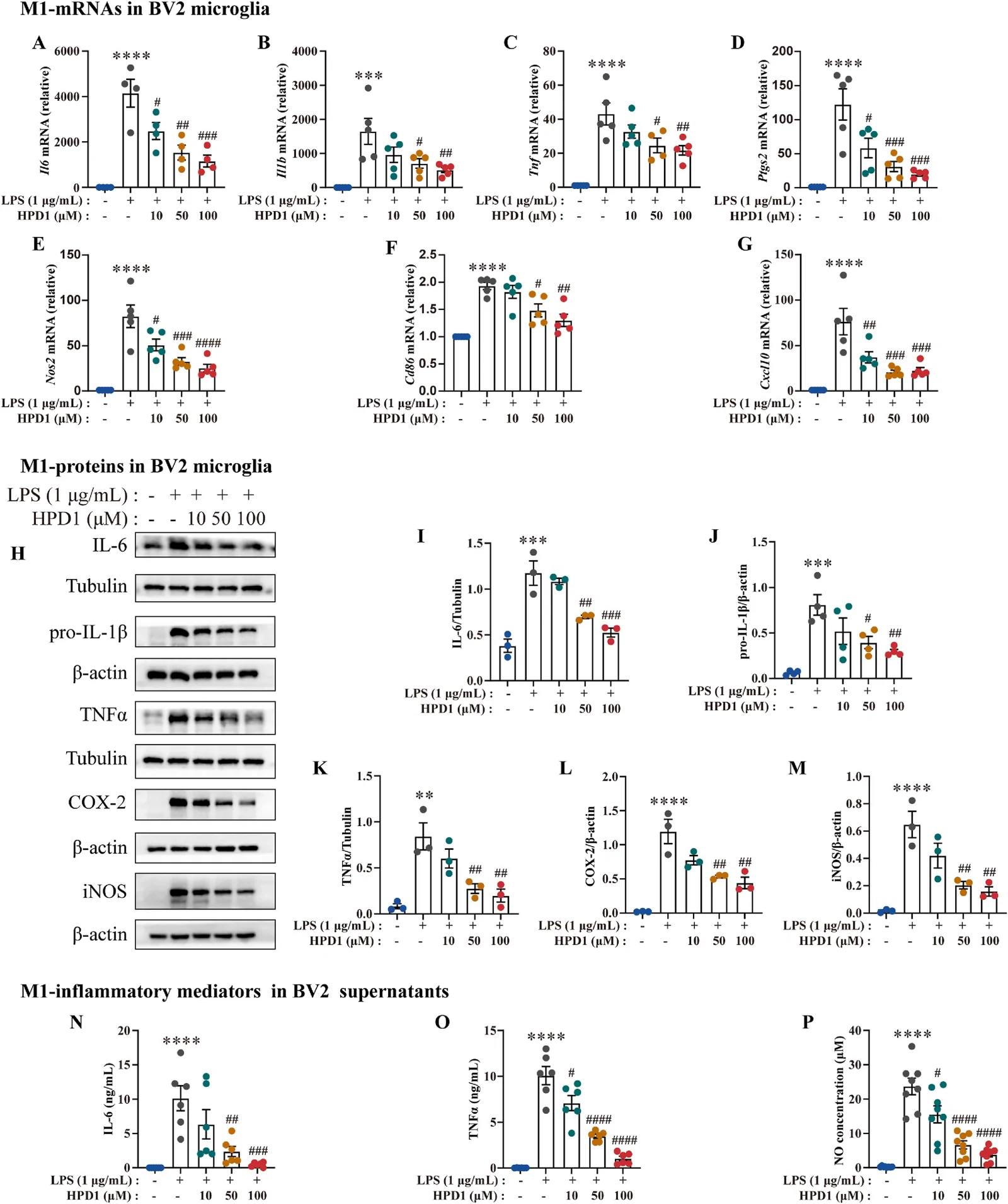
**HPD1 inhibited LPS-induced M1 polarization of BV2 microglia.** **(A-G)** Relative mRNA expression levels of M1 pro-inflammatory markers, including **(A)***Il6* (encoding IL-6), **(B)***Il1b* (IL-1β), **(C)***Tnf* (TNFα), **(D)***Ptgs2* (COX-2), **(E)***Nos2* (iNOS), **(F)***Cd86* (CD86), and **(G)***Cxcl10* (CXCL10) (n = 4 or 5). **(H)** Representative Western blot images showing the protein expression levels of IL-6, pro-IL-1β, TNFα, COX-2, and iNOS. **(I-M)** Quantitative analysis of the protein expression levels of **(I)** IL-6, **(J)** pro-IL-1β, **(K)** TNFα, **(L)** COX-2, and **(M)** iNOS (n = 3 or 4). **(N-P)** Concentrations of **(N)** IL-6, **(O)** TNFα, and **(P)** NO in cell culture supernatants (n = 6 or 8). Data are presented as mean ± SEM and analyzed using one-way ANOVA followed by Tukey's multiple comparisons test. **p < 0.01, ***p < 0.001, ****p < 0.0001 *vs.* Control group; #*p* < 0.05, ##*p* < 0.01, ###*p* < 0.001, ####*p* < 0.0001 *vs.* LPS-only group. CD86: cluster of differentiation 86; COX-2: cyclooxygenase-2; CXCL10: CXC motif chemokine ligand 10; iNOS: inducible nitric oxide synthase; IL-1β: interleukin-1β; IL-6: interleukin-6; LPS: lipopolysaccharide; NO: nitric oxide; TNFα: tumor necrosis factor-α.

### 3.4 HPD1 suppressed astrocytic A1-like polarization

To further assess whether the anti-inflammatory effect of HPD1 occurs in astrocytes, we first examined its impact on the expression of LPS-induced A1-type markers. Our results demonstrated that LPS stimulation markedly increased the protein and mRNA levels of C3 by 6.8-fold and 23.7-fold, respectively, as well as the mRNA level of *Serping1* (encoding C1-INH) by 6.3-fold. HPD1 treatment dose-dependently downregulated these A1-reactive markers, with 100 μM HPD1 suppressing C3 protein, *C3* mRNA, and *Serping1* mRNA levels by 40%, 37%, and 51%, respectively, relative to the LPS-only group (Fig. 4A–C). Subsequently, we investigated its effects on A1-associated pro-inflammatory mediators. LPS stimulation drastically upregulated the mRNA levels of key pro-inflammatory cytokines, including *Il6* (757.0-fold), *Il1b* (5220.1-fold), and *Tnf* (33.9-fold). Treatment with 100 μM HPD1 exhibited potent anti-inflammatory activity, significantly suppressing their gene transcription by 46%, 50%, and 68%, respectively (Fig. 4D–F). Consistently, ELISA assays confirmed that 100 μM HPD1 treatment markedly reduced the protein secretion of IL-6 by 56% (Fig. 4G) and TNFα by 83% (Fig. 4H) into the culture supernatants. Additionally, HPD1 treatment effectively attenuated iNOS expression at both the mRNA (Fig. 4I, *Nos2*, suppressed by 3.0-fold) and protein levels (Fig. 4J and K, suppressed by 5.2-fold), consequently reducing NO production by 4.1-fold (Fig. 4L). Taken together, these results demonstrated that HPD1 exerts significant anti-inflammatory effects by inhibiting astrocyte A1-like polarization.

**Fig. 4:**
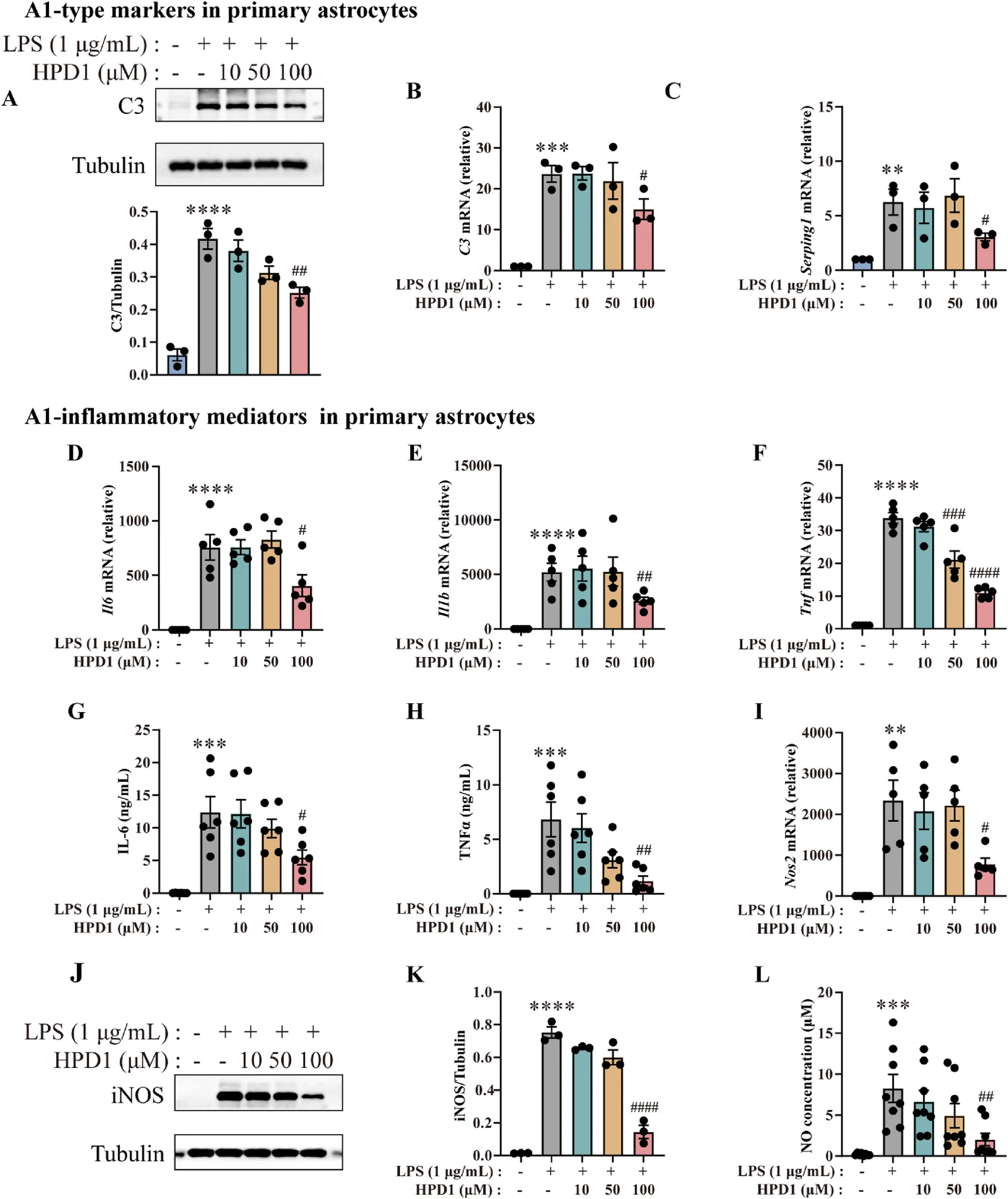
**HPD1 attenuated LPS-induced A1-like polarization of primary astrocytes.** **(A-C)** The expression levels of A1-type markers: **(A)** representative Western blot image and quantitative analysis of C3, and mRNA expression levels of **(B)***C3* (encoding C3) and **(C)***Serping1* (C1-INH) (n = 3). **(D-H)** The expression levels of pro-inflammatory cytokines: **(D-F)** mRNA expression levels of **(D)***Il6* (IL-6), **(E)***Il1b* (IL-1β), and **(F)** T*nf* (TNFα) (n = 5); **(G, H)** concentrations of **(G)** IL-6 and **(H)** TNFα in cell culture supernatants (n = 6). **(I-L)** iNOS/NO pathway: **(I)** mRNA expression levels of *Nos2* (iNOS) (n = 5); **(J, K)** representative Western blot image **(J)** and quantitative analysis **(K)** of iNOS (n = 3); **(L)** NO production in cell culture supernatants (n = 8). Data are presented as mean ± SEM and analyzed using one-way ANOVA followed by Tukey's multiple comparisons test. **p < 0.01, ***p < 0.001, ****p < 0.0001 *vs.* Control group; #*p* < 0.05, ##*p* < 0.01, ###*p* < 0.001, ####*p* < 0.0001 *vs.* LPS-only group. C1-INH: C1 inhibitor; C3: complement component 3; iNOS: inducible nitric oxide synthase; IL-1β: interleukin-1β; IL-6: interleukin-6; LPS: lipopolysaccharide; NO: nitric oxide; TNFα: tumor necrosis factor-α.

### 3.5 HPD1 promoted microglial M2 and astrocytic A2-like polarization

To further clarify whether HPD1 promotes the phenotypic transition of glial cells toward a protective state, we examined its effects on the mRNA and protein expression levels of M2 microglial and A2 astrocytic markers. First, RT-qPCR analysis showed that LPS stimulation significantly suppressed the mRNA expression of M2 microglial markers, including *Tgfb* (encoding TGFβ, downregulated by 2.8-fold), *Mrc1* (CD206, 5.7-fold), and Arg*1* (ARG1, 12.0-fold) (Fig. 5A–C). Upon co-treatment with LPS, HPD1 dose-dependently restored the mRNA levels of *Tgfb* by 2.7-fold (100 μM) and *Mrc1* by 1.7-fold (100 μM) (Fig. 5A and B), whereas Arg*1* expression remained unaltered by HPD1 treatment (Fig. 5C). Given that ARG1 and iNOS compete for L-arginine, the essential substrate for NO synthesis, this decreased iNOS/ARG1 ratio functionally accounts for the diminished NO production observed following HPD1 treatment. For *Il10* (IL-10), LPS stimulation did not significantly affect its mRNA expression. HPD1 treatment, however, dose-dependently upregulated its expression by 3.8-fold (Fig. 5D, 100 μM). Subsequently, we evaluated the expression of A2 astrocytic markers in primary astrocytes. The results indicated that LPS stimulation markedly suppressed the protein and mRNA levels of S100A10 (a 2.0-fold and 2.4-fold decrease, respectively) and the mRNA level of *Cd109* (CD109, a 4.1-fold reduction). Treatment with 100 μM HPD1 effectively restored the expression of S100A10 protein, *S100a1*0 mRNA, and *Cd1*09 mRNA levels by 1.8-fold, 2.2-fold, and 2.1-fold, respectively, compared to the LPS-only group (Fig. 5E–H). For *Clcf1* (CLCF1), LPS stimulation did not significantly affect its mRNA expression, while treatment with 100 μM HPD1 significantly upregulated its expression by 1.5-fold (Fig. 5I). Collectively, these findings suggested that HPD1 exerts potent anti-inflammatory effects by promoting neuroprotective M2 microglial and A2-like astrocytic phenotypes.

**Fig. 5:**
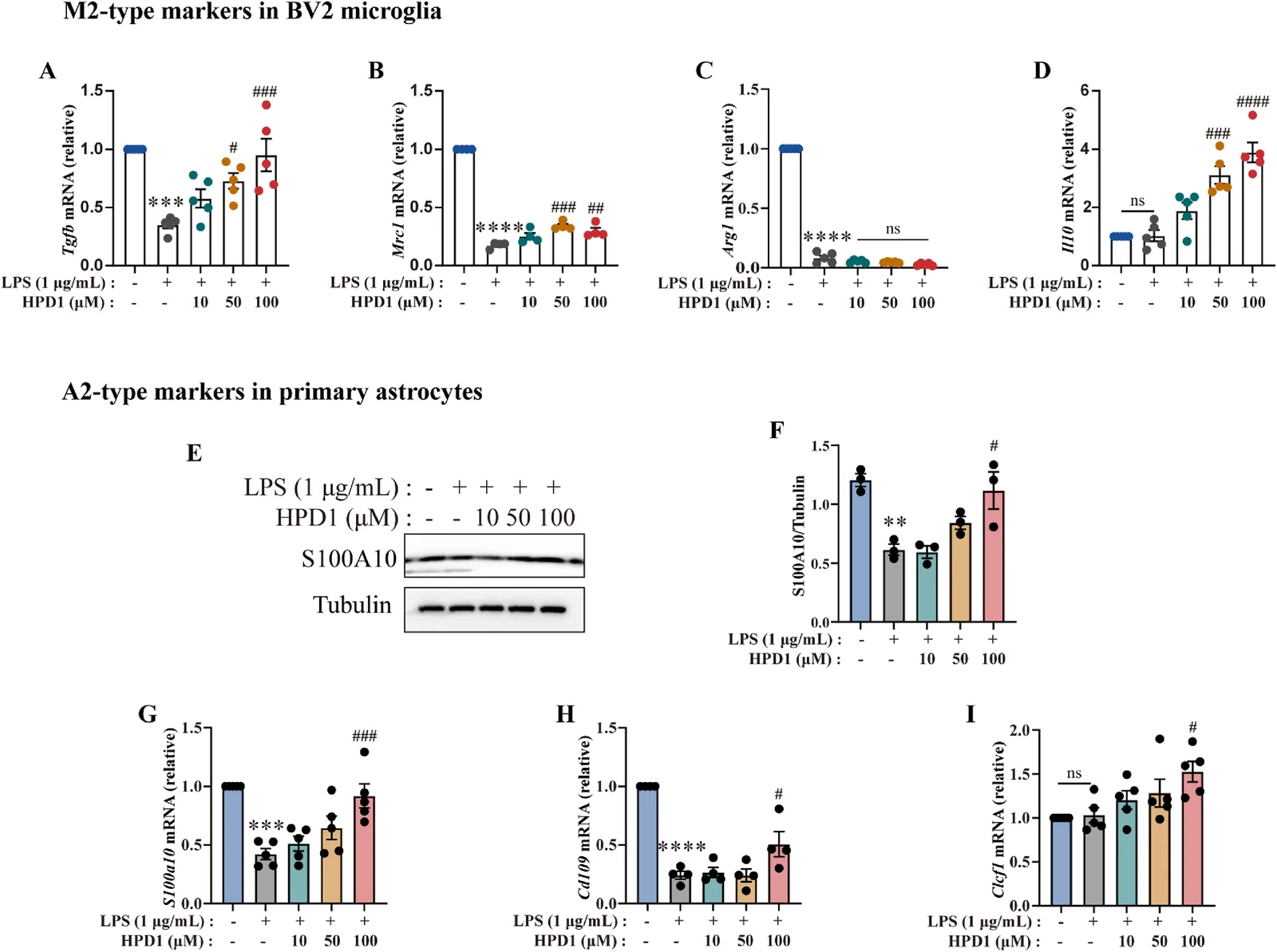
**HPD1 promoted M2 polarization of BV2 microglia and A2-like polarization of primary astrocytes.** **(A-D)** Relative mRNA expression levels of M2-type anti-inflammatory markers in BV2 microglia, including **(A)***Tgfb* (encoding TGFβ), **(B)***Mrc1* (CD206), **(C)** Arg*1* (ARG1), and **(D)***Il10* (IL-10) (n = 4 or 5). **(E-F)** Representative Western blot image **(E)** and quantitative analysis **(F)** showing the protein expression level of S100A10 in primary astrocytes (n = 3). **(G-I)** Relative mRNA expression levels of A2-type markers in primary astrocytes, including **(G)***S100a10* (S100A10), **(H)***Cd109* (CD109), and **(I)***Clcf1* (CLCF1) (n = 4 or 5). Data are presented as mean ± SEM and analyzed using one-way ANOVA followed by Tukey's multiple comparisons test. **p < 0.01, ***p < 0.001, ****p < 0.0001 *vs.* Control group; #*p* < 0.05, ##*p* < 0.01, ###*p* < 0.001, ####*p* < 0.0001 *vs.* LPS-only group; ns, not significant. ARG1: arginase-1; CD109: cluster of differentiation 109; CD206: macrophage mannose receptor; CLCF1: cardiotrophin-like cytokine factor 1; IL-10: interleukin-10; LPS: lipopolysaccharide; S100A10: S100 calcium-binding protein A10; TGFβ: transforming growth factor-β.

### 3.6 HPD1 inhibited TLR4-MAPK/NF-κB pathway in the LPS-stimulated BV2 cells

To elucidate the molecular mechanisms underlying the anti-inflammatory effects of HPD1 in BV2 cells, we focused on the canonical TLR4-MAPK/NF-κB signaling pathway (Fig. 6A). Western blot analysis demonstrated that LPS stimulation markedly upregulated TLR4 expression (35.7-fold) and the phosphorylation levels of key components of MAPK pathway, including p-ERK1/2/ERK1/2 (4.7-fold), p-p38/p38 (2.1-fold), p-JNK/JNK (3.0-fold), p-IκBα/IκBα (1.8-fold), and p-p65/p65 (13.9-fold). HPD1 treatment dose-dependently suppressed the LPS-induced upregulation of these proteins. Specifically, 100 μM HPD1 significantly reduced TLR4 expression by 56% (Fig. 6B), p-ERK1/2/ERK1/2 by 58% (Fig. 6C), p-p38/p38 by 34% (Fig. 6D), and p-JNK/JNK by 60% (Fig. 6E). Furthermore, 100 μM HPD1 effectively inhibited NF-κB activation by decreasing p-IκBα/IκBα (Fig. 6F) and p-p65/p65 (Fig. 6G) levels by 64% and 73%, respectively, relative to the LPS-only group. Additionally, immunofluorescence staining further confirmed that HPD1 treatment inhibited the nuclear translocation of p65, maintaining it primarily within the cytoplasm compared with the LPS-only group (Fig. 6H). Collectively, these results indicated that HPD1 exerts its anti-inflammatory effects by suppressing the TLR4-MAPK/NF-κB signaling pathway.

**Fig. 6:**
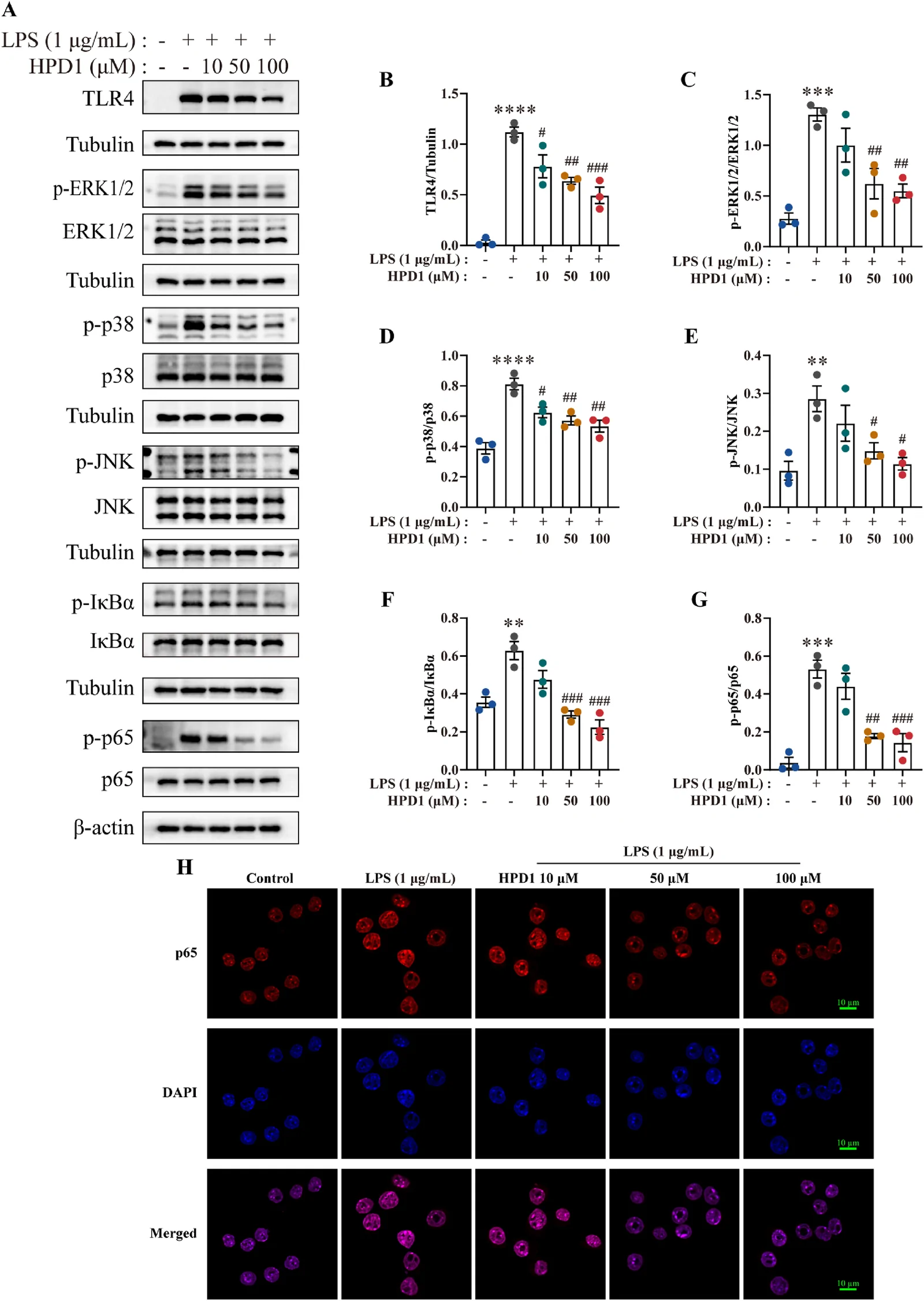
**HPD1 inhibited the activation of TLR4-MAPK/NF-κB pathway in BV2 microglia.** **(A)** Representative Western blot images showing the phosphorylation and total protein levels of TLR4, p-ERK/ERK, p-p38/p38, p-JNK/JNK, p-IκBα/IκBα, and p-p65/p65. **(B-G)** Quantitative analysis of the protein expression levels of **(B)** TLR4, **(C)** p-ERK/ERK, **(D)** p-p38/p38, **(E)** p-JNK/JNK, **(F)** p-IκBα/IκBα, and **(G)** p-p65/p65 (n = 3). **(H)** Representative immunofluorescence images showing the nuclear translocation of p65 (red). Nuclei were stained with DAPI (blue), scale bar = 10 μm (n = 3). Data were presented as mean ± SEM and analyzed using one-way ANOVA followed by Tukey's multiple comparisons test. **p < 0.01, ***p < 0.001, ****p < 0.0001 *vs.* Control group; #*p* < 0.05, ##*p* < 0.01, ###*p* < 0.001 *vs.* LPS-only group. ERK1/2: extracellular signal-regulated kinase 1 and 2; IκBα: inhibitor of κBα; JNK: c-Jun N-terminal kinase; LPS: lipopolysaccharide; MAPK: mitogen-activated protein kinase; NF-κB: nuclear factor-κB; p-: phosphorylated; TLR4: toll-like receptor 4.

### 3.7 HPD1 suppressed NF-κB pathway in the LPS-stimulated primary astrocytes

To further investigate the anti-inflammatory mechanism of HPD1 in primary astrocytes, we analyzed its effects on the activation of the TLR4-MAPK/NF-κB signaling pathway. Western blot analysis revealed that LPS challenge robustly activated the canonical NF-κB signaling pathway, inducing an 8.0-fold increase in IκBα phosphorylation (Fig. 7A and B) and triggering its subsequent degradation (reflected by a 1.9-fold decrease in total IκBα expression) (Fig. 7C and D); concurrently, the phosphorylation ratio of p-p65/p65 was markedly elevated by 4.6-fold (Fig. 7E and F). Compared with the LPS-only group, 100 μM HPD1 treatment inhibited IκBα phosphorylation by 1.5-fold and prevented its degradation, increasing total IκBα protein level by 2.1-fold (Fig. 7A–D). Meanwhile, 100 μM HPD1 significantly suppressed p65 phosphorylation, yielding a 1.8-fold decrease in the p-p65/p65 ratio (Fig. 7E and F). Interestingly, no significant changes were observed in TLR4 protein expression and MAPK cascade activation (ERK1/2, p38, and JNK) across all groups (Fig. 7G–K), as the LPS-activated TLR4-MAPK signaling pathway had largely returned to baseline levels at this late stage of the inflammatory response. Collectively, these findings demonstrated that HPD1 effectively suppresses LPS-induced inflammatory responses in primary astrocytes by inhibiting the canonical NF-κB signaling pathway.

**Fig. 7:**
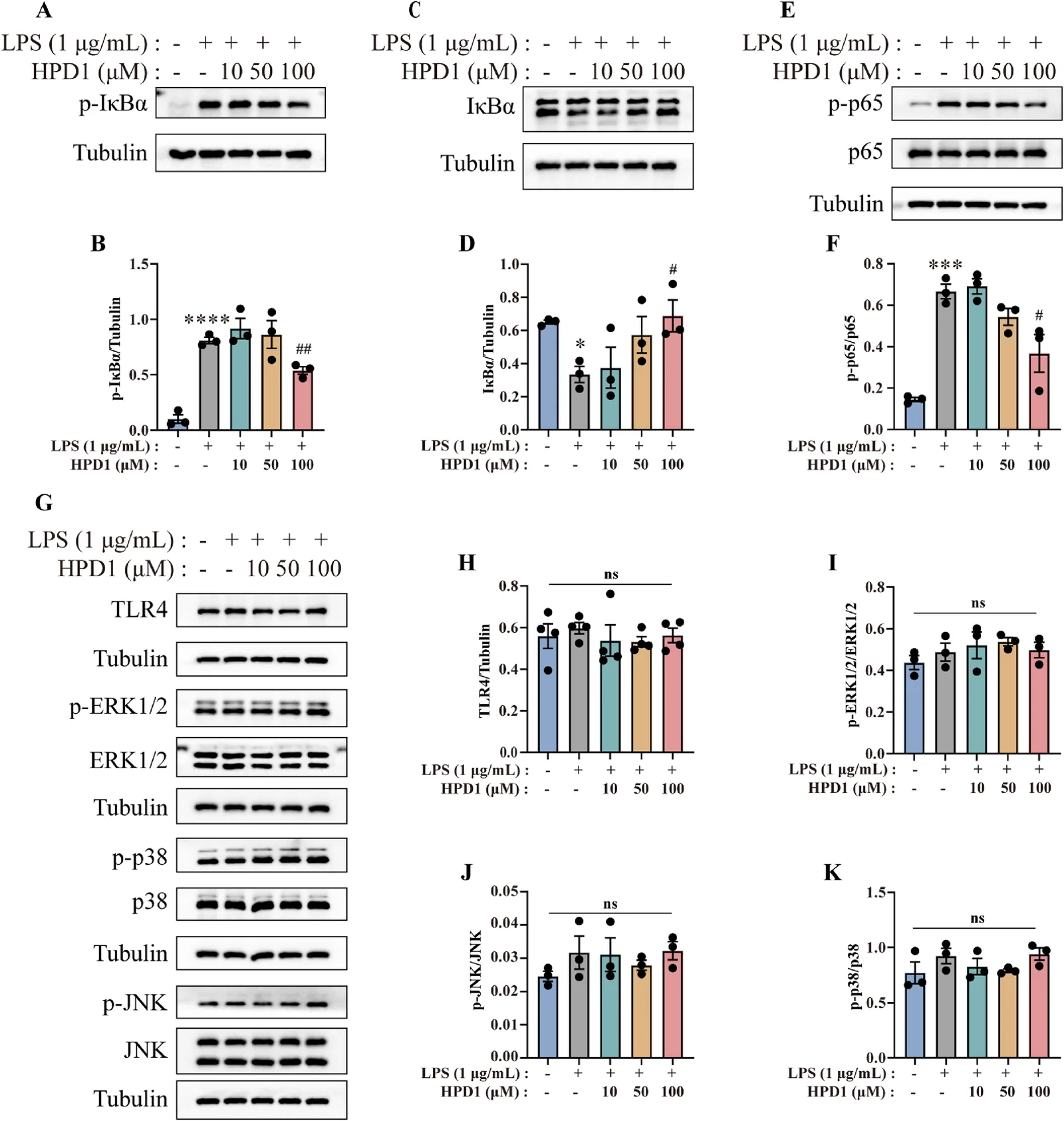
**HPD1 inhibited the activation of NF-κB signaling, but not TLR4-MAPK pathway, in primary astrocytes.** **(A, B)** Representative Western blot image **(A)** and quantitative analysis **(B)** of p-IκBα (n = 3). **(C, D)** Representative Western blot image **(C)** and quantitative analysis **(D)** of IκBα (n = 3). **(E, F)** Representative Western blot image **(E)** and quantitative analysis **(F)** of p-p65/p65 (n = 3). **(G)** Representative Western blot images showing the protein expression levels of TLR4-MAPK signaling molecules. **(H-K)** Quantitative analysis of **(H)** TLR4, **(I)** p-ERK1/2/ERK1/2, **(J)** p-JNK/JNK, and (K) p-p38/p38 (n = 3). Data are presented as mean ± SEM and analyzed using one-way ANOVA followed by Tukey's multiple comparisons test. *p < 0.05, ***p < 0.001, ****p < 0.0001 *vs.* Control group; #*p* < 0.05, ##*p* < 0.01 *vs.* LPS-only group; ns, not significant. ERK1/2: extracellular signal-regulated kinase 1 and 2; IκBα: inhibitor of κBα; JNK: c-Jun N-terminal kinase; LPS: lipopolysaccharide; MAPK: mitogen-activated protein kinase; NF-κB: nuclear factor-κB; p-: phosphorylated; TLR4: Toll-like receptor 4.

### 3.8 HPD1 conferred neuroprotection against BV2 microglial CM-induced neurotoxicity

To evaluate whether HPD1-mediated inhibition of microglial activation protects neurons against neurotoxicity, HT22 neuronal cells were treated with CM derived from LPS-activated BV2 microglia (LPS-CM). The MTT assay demonstrated that exposure to LPS-CM significantly impaired cell viability, whereas treatment with HPD1-treated BV2 microglia CM (HPD1-CM) concentration-dependently restored HT22 cell viability (Fig. 8A; *F*_(4, 20)_ = 10.92, *p* < 0.0001). Consistent with these findings, brightfield microscopy revealed severe morphological damage in LPS-CM-treated HT22 cells, characterized by marked loss of cell density, soma shrinkage, and neurite retraction, which was substantially rescued by HPD1-CM (50 μM and 100 μM) (Fig. 8B). The Annexin V/PI flow cytometry results further demonstrated that LPS-CM exposure markedly increased the neuronal apoptotic rate by 3.1-fold compared with the Control group. Treatment with HPD1-CM dose-dependently rescued neurons from apoptosis, with 100 μM HPD1-CM yielding a 2.2-fold decrease in the apoptotic rate relative to the LPS-CM group (Fig. 8C and D; *F*_(4, 10)_ = 38.62, *p* < 0.0001). Consistently, Calcein-AM/PI live/dead staining confirmed that LPS-CM significantly increased neurotoxic cell death (Fig. 8E), whereas HPD1-CM treatment strikingly suppressed this cell death as shown by reduced dead cell counts (Fig. 8F; *F*_(4, 10)_ = 110.7, *p* < 0.0001) and lower percentages of dead cell (Fig. 8G; *F*_(4, 10)_ = 78.73, *p* < 0.0001). Collectively, these results demonstrated that HPD1 protects against microglial CM-induced neurotoxicity and exhibits potent neuroprotective properties.

**Fig. 8:**
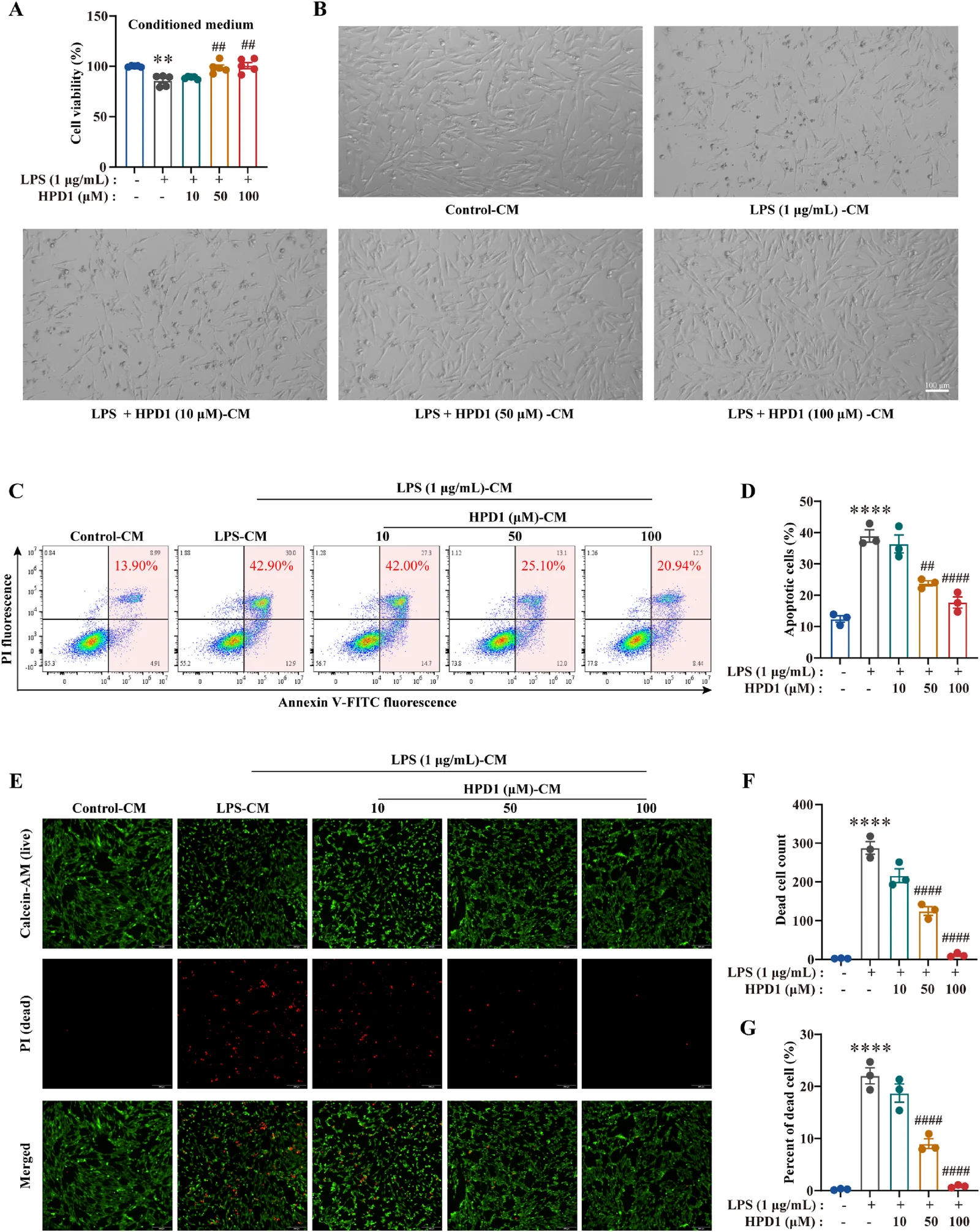
**HPD1 protected HT22 cells against BV2 microglial CM-induced neurotoxicity.** **(A)** HT22 cell viability measured by MTT assay after treatment with CM derived from LPS-stimulated BV2 microglia with or without HPD1 (n = 5). **(B)** Representative brightfield images showing the morphological changes in HT22 cells, scale bar = 100 μm. **(C, D)** Representative flow cytometry scatter plots **(C)** and quantitative analysis **(D)** of apoptotic HT22 cells stained with Annexin V-FITC/PI (n = 3). **(E-G)** Representative fluorescence images **(E)** and quantitative analysis of dead cell count **(F)** and the percentage of dead cell **(G)** stained with Calcein-AM (green, live cells) and PI (red, dead cells), scale bar = 200 μm (n = 3). Data are presented as mean ± SEM and analyzed using one-way ANOVA followed by Tukey's multiple comparisons test. **p < 0.01, ****p < 0.0001 *vs*. Control group; ##*p* < 0.01, ####*p* < 0.0001 *vs*. LPS-only group. CM: conditioned medium; FITC: fluorescein isothiocyanate; LPS: lipopolysaccharide; MTT: 3-(4,5-dimethylthiazol-2-yl)-2,5-diphenyltetrazolium bromide; PI: propidium iodide.

### 3.9 HPD1 reduced serum pro-inflammatory cytokine levels in mouse models

To preliminarily evaluate the in vivo anti-inflammatory efficacy of HPD1, serum cytokine levels were measured using ELISA in both acute neuroinflammation and chronic neuropathic pain mouse models. In the acute spinal neuroinflammation model induced by intrathecal LPS injection (Fig. 9A), serum levels of pro-inflammatory cytokines were markedly elevated 2 h post-injection. Co-administration of HPD1 significantly suppressed systemic inflammation in a dose-dependent manner. Specifically, high-dose HPD1 dramatically reduced serum IL-6 levels by 1.6-fold and TNFα levels by 2.2-fold relative to the LPS-only group (Fig. 9B and C). Furthermore, we assessed the systemic anti-inflammatory effect of HPD1 in the CCI-induced neuropathic pain model (Fig. 9D). On post-operative day 7, CCI mice exhibited significant elevations in serum IL-6, IL-1β, and TNFα levels compared with Sham-operated mice. A single intraperitoneal injection of HPD1 suppressed systemic inflammation after 2 h, significantly decreasing serum IL-6, IL-1β, and TNFα levels by 92%, 66%, and 65%, respectively, relative to the CCI group (Fig. 9E–G). Together, these results demonstrated that HPD1 effectively suppresses systemic pro-inflammatory cytokine production in both acute and chronic neuroinflammatory conditions in vivo.

**Fig. 9:**
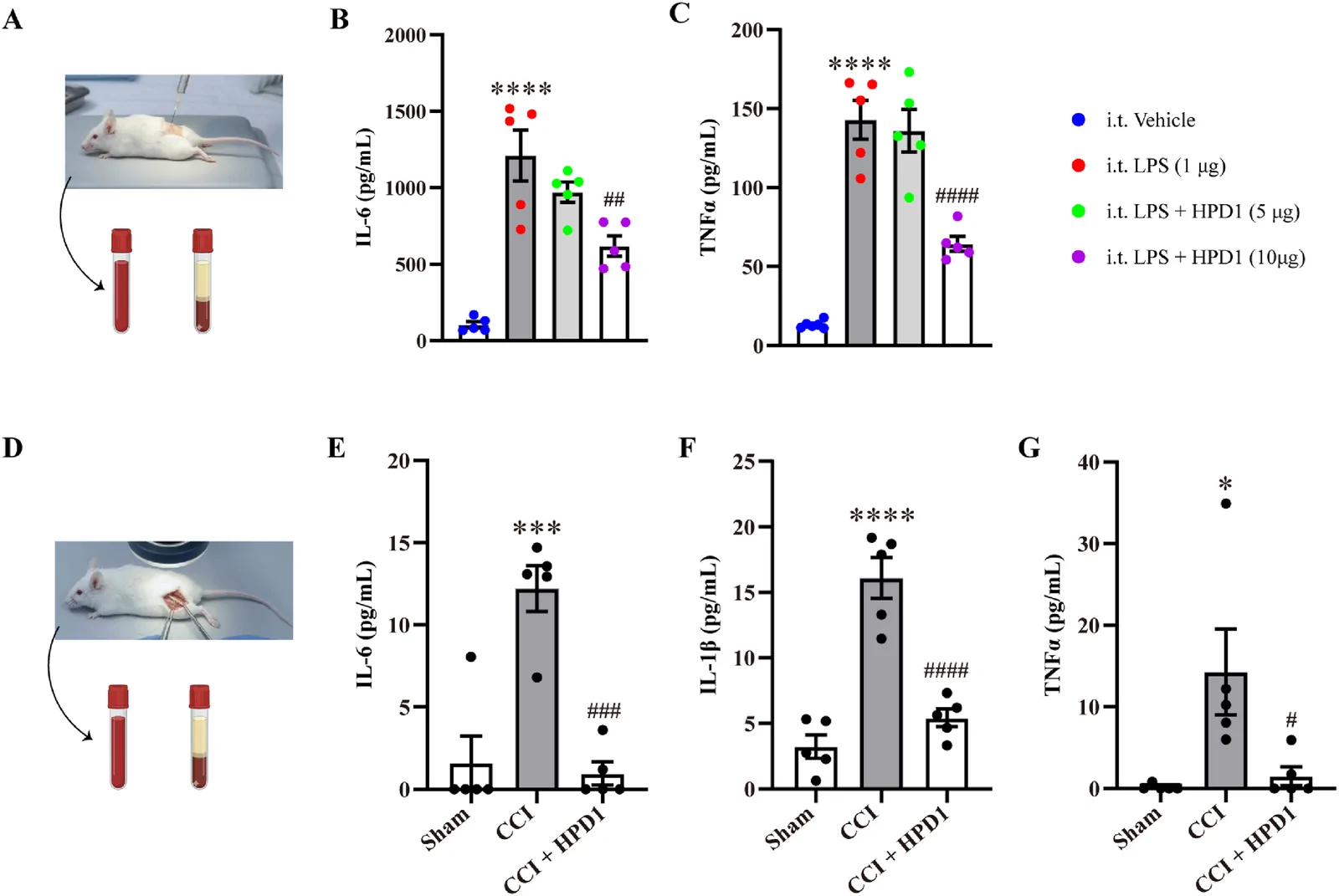
**HPD1 inhibited pro-inflammatory cytokine production in mouse models of acute neuroinflammation and chronic neuropathic pain.** **(A)** Schematic representation of the intrathecal LPS-induced acute neuroinflammation model. **(B, C)** Serum levels of **(B)** IL-6 and **(C)** TNFα measured by ELISA 2 h after intrathecal co-administration of LPS and HPD1 (n = 5). **(D)** Schematic diagram of the sciatic nerve CCI model. **(E-G)** Serum levels of **(E)** IL-6, **(F)** IL-1β, and **(G)** TNFα measured by ELISA 2 h after a single intraperitoneal injection of HPD1 in CCI mice on post-operative day 7 (n = 5). Data were presented as mean ± SEM and analyzed using one-way ANOVA followed by Tukey's multiple comparisons test. *p < 0.05, ***p < 0.001, ****p < 0.0001 *vs*. Vehicle or Shan group; #*p* < 0.05, ##*p* < 0.01, ###*p* < 0.001, ####*p* < 0.0001 *vs*. LPS-only or CCI group. CCI: chronic constriction injury; IL-1β: interleukin-1β; IL-6: interleukin-6; i.t.: intrathecal; LPS: lipopolysaccharide; TNFα: tumor necrosis factor-α.

## 4 Discussion

Neuroinflammation serves as a protective defense mechanism for the CNS by clearing endogenous and exogenous pathological insults; however, sustained chronic inflammatory responses are highly destructive [8,43]. Growing evidence indicates that RSS deficiency is a key determinant of chronic neuroinflammation, contributing to aberrant glial activation and the pathological progression of various CNS disorders [1,2]. Here, we demonstrated that the novel organic H_2_S_2_ donor HPD1 exhibited superior cytoprotective and anti-inflammatory efficacy compared with inorganic H_2_S and H_2_S_2_ donors in LPS-stimulated BV2 microglia. Notably, the dose-response profile of its bioactivity closely matched the intracellular H_2_S_2_ accumulation in BV2 microglia, suggesting that the pharmacological effects of HPD1 are most likely mediated by the released H_2_S_2_ rather than the parent molecule itself.

Biologically, H_2_S_2_ supplementation via HPD1 effectively modulated glial phenotypic polarization in LPS-stimulated BV2 microglia and primary astrocytes, thereby suppressing excessive neuroinflammation and conferring neuroprotection (summarized in Fig. 10). Specifically, HPD1 dose-dependently attenuated LPS-induced cytotoxicity and shifted microglia and primary astrocytes from pro-inflammatory, neurotoxic states toward anti-inflammatory, neuroprotective phenotypes, as characterized by M1/M2 and A1/A2 marker transitions, respectively. Mechanistically, HPD1 exerted its anti-inflammatory and neuroprotective effects by suppressing the TLR4-MAPK/NF-κB signaling in BV2 microglia, whereas in primary astrocytes, it predominantly inhibited the NF-κB signaling pathway, thereby protecting neuronal HT22 cells against neurotoxicity induced by CM from LPS-activated BV2 microglia. Consistently, in vivo evaluation further confirmed that HPD1 robustly suppressed systemic pro-inflammatory cytokine release in mouse models of both acute LPS-induced neuroinflammation and chronic CCI neuropathic pain.

**Fig. 10:**
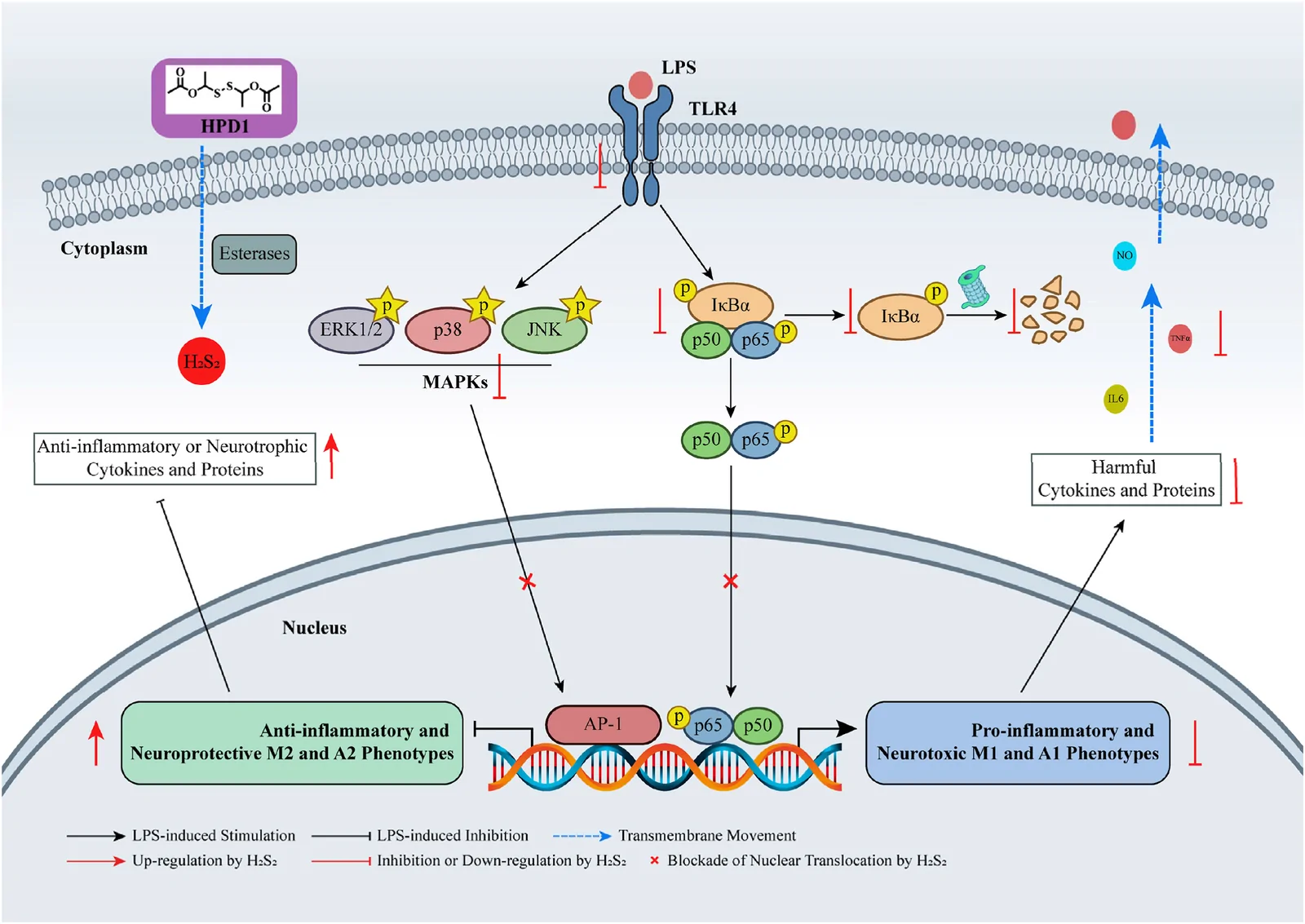
**Schematic diagram illustrating the mechanisms of HPD1-mediated phenotypic reprogramming.** HPD1, an esterase-responsive H_2_S_2_ donor, orchestrates a phenotypic shift in LPS-stimulated BV2 microglia and primary astrocytes, transitioning them from a pro-inflammatory, neurotoxic state toward an anti-inflammatory, neuroprotective phenotype by inhibiting the TLR4-MAPK/NF-κB signaling pathway, thereby protecting neurons from inflammatory injury. AP-1: activator protein 1; ERK1/2: extracellular signal-regulated kinase 1 and 2; H_2_S_2_: hydrogen persulfide; IκBα: inhibitor of κBα; IL-6: interleukin-6; JNK: c-Jun N-terminal kinase; MAPK: mitogen-activated protein kinase; NF-κB: nuclear factor-κB; NO: nitric oxide; p: phosphorylation; TLR4: toll-like receptor 4; TNFα: tumor necrosis factor-α.

In response to persistent pathological insults, microglia and astrocytes are activated and exhibit remarkable morphological and functional plasticity [44,45]. Under pathological conditions, these glial cells typically undergo hypertrophy [46], with microglia displaying a characteristic amoeboid morphology [47]. Our results demonstrated that HPD1 significantly attenuated the LPS-induced amoeboid transformation of BV2 microglia, promoting a reversion toward a more homeostatic morphology. This morphological improvement is closely linked to a shift in functional phenotype. Activated BV2 microglia manifest two distinct polarizations: the pro-inflammatory and neurotoxic M1 phenotype, and the anti-inflammatory and neuroprotective M2 phenotype [48]. LPS-induced M1 microglia initiate the expression of a vast array of inflammatory mediators including inflammatory proteins, chemokines, and pro-inflammatory cytokines, thereby creating a pro-inflammatory microenvironment that exerts significant cytotoxicity toward adjacent astrocytes and neurons [48]. Our results demonstrated that HPD1 significantly downregulated the expression of CD86, COX-2, and iNOS at the gene or protein levels. As iNOS and COX-2 are the key rate-limiting enzymes driving the production of NO and prostaglandins, respectively, their downregulation mediated by HPD1 directly decreased the cellular capacity to generate oxidative stress and inflammatory response. Furthermore, HPD1 downregulated the mRNA expression of CXCL10 and reduced the secretion of pro-inflammatory cytokines (IL-6 and TNFα) as well as the release of NO, thereby mitigating neurotoxicity toward neuronal HT22 cells. Although the primary responsibility of astrocytes is to maintain neuronal homeostasis and provide metabolic and neurotrophic support, activated astrocytes are intimately involved in the orchestration of neuroinflammation [49]. Consistent with microglia, LPS-stimulated astrocytes manifest distinct neurotoxic A1-like and neuroprotective A2-like phenotypes. Our findings revealed that HPD1 treatment significantly downregulated the expression of A1 markers C3 and C1-INH, thereby inhibiting A1-like polarization and the subsequent production of inflammatory mediators. Specifically, HPD1 markedly reduced the mRNA expression of IL-6, IL-1β, and TNFα, as well as the secretory release of IL-6 and TNFα, alongside the suppression of the iNOS/NO pathway, which also likely served to reduce potential neuronal injury. Beyond its potent inhibitory effects on M1 and A1-like polarization, HPD1 exerted its anti-inflammatory and neuroprotective effects by actively promoting M2 and A2-like phenotypes. Our results demonstrated that HPD1 treatment markedly upregulated M2 markers (TGFβ, CD206, and IL-10) and A2 markers (S100A10, CD109, and CLCF1), indicating a profound shift toward neuroprotective glial phenotypes. Collectively, these findings indicate that HPD1 reprograms the balance of phenotypic polarization in both microglia and astrocytes to combat neuroinflammation, thereby conferring neuroprotection and restoring neuroimmune homeostasis.

NF-κB and activator protein 1 (AP-1) serve as essential transcription factors for the expression of inflammatory mediators in activated microglia and astrocytes [[50], [51], [52], [53]]. Upon recognition and binding of LPS to the cell membrane-bound TLR4 receptor, the downstream MAPK and NF-κB signaling pathways are rapidly initiated via TGFβ activated kinase 1 activation, thereby triggering the transcriptional activity of NF-κB and AP-1, which leads to the robust expression and release of various inflammatory mediators [52,53]. Our results demonstrated that HPD1 downregulated the protein expression of TLR4 in response to LPS in BV2 microglia, potentially by limiting the activation of MAPK and NF-κB signaling pathways. The MAPKs, which primarily include the three key enzymes ERK1/2, p38, and JNK, are involved in complex signaling pathways due to the presence of numerous cytoplasmic and nuclear substrates [54]. Upon LPS stimulation, TLR4 mediates the phosphorylation and activation of MAPKs, which then translocate into the nucleus to phosphorylate the transcription factor AP-1 comprising c-Jun and c-Fos, thereby initiating the expression of various inflammatory mediators [52,53]. Our study demonstrated that HPD1 markedly attenuated the phosphorylation of ERK1/2, p38, and JNK, leading to the potential inhibition of their nuclear translocation and the downstream AP-1 transcriptional activity in BV2 cells. Interestingly, this inhibitory effect on the TLR4-MAPK signaling pathway was not observed in primary astrocytes following 24 h of LPS challenge, as the activation of TLR4-MAPK components had already returned to basal levels by this prolonged time point [55], likely driven by the engagement of endogenous negative feedback loops. This temporal discrepancy highlights distinct signaling kinetics between microglia and astrocytes, suggesting that HPD1 most likely acts within an early activation window or may modulate astrocyte responses through alternative redox-sensitive mechanisms.

NF-κB typically works in synergy with the AP1 transcription factor to initiate the expression of inflammatory mediators [52,53,56]. Under physiological conditions, NF-κB is sequestered in the cytoplasm by inhibitory IκB proteins. Upon stimulation by LPS, IκB proteins are phosphorylated and undergo rapid degradation through the ubiquitin-proteasome pathway. This triggers the release and translocation of the NF-κB p65/p50 heterodimer into the nucleus, where it initiates the transcription of inflammatory mediators [13]. Our results revealed that HPD1 hindered the transcriptional activity of NF-κB in BV2 microglial cells and primary astrocytes. In both cell types, HPD1 inhibited IκBα phosphorylation and reduced p65 phosphorylation. In primary astrocytes, HPD1 also prevented IκBα degradation and restored total IκBα protein levels, whereas in BV2 microglia, HPD1 blocked the nuclear translocation of p65. Moreover, the downstream transcriptional products of NF-κB and AP-1, including TLR4, TNFα, IL-1β, and others, in turn reverse-activate NF-κB and AP-1, which establishes a positive feedback loop that amplifies inflammatory signals. Inhibition of the NF-κB signaling pathway by HPD1 effectively interrupted this self-amplifying inflammatory cascade. Collectively, HPD1 exerts its robust anti-inflammatory effects in BV2 microglial cells and primary astrocytes by suppressing the NF-κB signaling pathway, thereby orchestrating a functional transition from pro-inflammatory M1 and A1-like states to neuroprotective M2 and A2-like phenotypes.

Consistently, the potent anti-inflammatory capacity of HPD1 observed in vitro translated directly into robust therapeutic efficacy in vivo. In the acute LPS-induced neuroinflammation model, intrathecal administration of HPD1 remarkably blunted systemic inflammatory responses, suppressing the surge of serum pro-inflammatory cytokines, such as IL-6 and TNFα. Additionally, chronic neuropathic pain is increasingly recognized as a form of gliopathy driven by dysregulated glial activity, which induces pain hypersensitivity through both peripheral and central sensitization [57]. In the chronic CCI-induced neuropathic pain model, systemic intraperitoneal administration of HPD1 significantly attenuated cold hypersensitivity and mechanical allodynia as previously reported [33], which in the present study was accompanied by a marked reduction in circulating levels of key pro-inflammatory cytokines, including IL-6, IL-1β, and TNFα. Collectively, these findings confirm the robust in vivo anti-inflammatory effects of HPD1, establishing organic H_2_S_2_ donors as promising candidates for treating inflammation-driven neurological and nociceptive disorders.

Indeed, HPD1 displayed dose-dependent variations in pharmacological efficacy between BV2 microglia and primary astrocytes. This variance likely originates from distinct inflammatory response profiles and differential cellular esterase expression profiles, which lead to heterogeneous intracellular HPD1 or H_2_S_2_ responsiveness. Although HPD1 effectively exerted robust immunomodulatory actions at 100 μM in the both cell types, these simplified cell culture systems cannot fully recapitulate the complex cellular and molecular interactions present in the intact CNS. Accordingly, while these in vitro findings demonstrated a broad anti-inflammatory profile for HPD1, the mechanistic impact of HPD1 on coordinated microglial-astrocytic-neuronal crosstalk remains to be further validated in vivo. Additionally, H_2_S_2_ released from HPD1 may be metabolized in vivo into other low-molecular-weight RSS, including H_2_S, cysteine persulfide, and glutathione persulfide. However, NaHS, an inorganic H_2_S donor, showed no significant pharmacological activity in our study, suggesting that the observed bioactivity of HPD1 is largely independent of its H_2_S metabolite. Although the present study did not perform comprehensive profiling of these low-molecular-weight RSS metabolites post-HPD1 treatment, and thus we cannot completely rule out the possibility that other downstream metabolites of H_2_S_2_ contribute to the observed biological effects, we nevertheless propose that H_2_S_2_ itself most probably serves as the primary driver. This is supported by its exceptional biochemical reactivity, which enables direct protein persulfidation to efficiently mediate downstream signaling cascades. Therefore, future investigations into the specific persulfidated proteins are highly warranted to fully elucidate the underlying molecular targets. In the present study, although we preliminarily demonstrated the systemic anti-inflammatory actions of HPD1 in acute neuroinflammatory and chronic neuropathic pain models, direct quantification of central H_2_S_2_ levels was not performed. Given its high chemical reactivity and rapid conversion in peripheral tissues and circulation, peripheral H_2_S_2_ generated by HPD1 is unlikely to cross the blood-brain barrier (BBB) substantially. Rather, HPD1 appears to act as a lipophilic prodrug that readily penetrates the BBB to exert central therapeutic effects, a concept supported by its optimal physicochemical properties for passive BBB permeation [58].

In conclusion, this study identifies the novel organic donor HPD1 as a highly effective H_2_S_2_ supplementing agent that alleviates glial neuroinflammation both in vitro and systemic inflammation in vivo. Mechanistically, HPD1 inhibits the canonical TLR4-MAPK/NF-κB in microglia and NF-κB signaling in astrocytes, thereby reprogramming M1/M2 and A1/A2 phenotypic balances to confer robust neuroprotection and restore neuroimmune homeostasis, positioning H_2_S_2_ supplementation as a promising therapeutic strategy for neuroinflammation-related diseases.

## CRediT authorship contribution statement

**Fei Yan:** Conceptualization, Data curation, Formal analysis, Investigation, Methodology, Validation, Writing – original draft, Writing – review & editing. **Shouwei Tao:** Data curation, Formal analysis, Investigation, Validation, Writing – review & editing. **Yan Jiao:** Funding acquisition, Methodology, Resources, Writing – review & editing. **Ting Kang:** Project administration, Resources, Writing – review & editing. **Shuangqing Du:** Resources, Writing – review & editing. **Yue Cheng:** Methodology, Writing – review & editing. **Zhenbo Huang:** Conceptualization, Supervision, Writing – original draft, Writing – review & editing. **Ping Feng:** Conceptualization, Methodology, Project administration, Supervision, Writing – review & editing. **Bowen Ke:** Conceptualization, Funding acquisition, Methodology, Project administration, Supervision, Writing – review & editing.

## Funding sources

This work was supported by grants from 10.13039/501100001809National Natural Science Foundation of China (82273784 and 82425054), Brain Science and Brain-like Intelligence Technology-National Science and Technology Major Project (2025ZD0214904, China), Innovation Drug Research and Development National Science and Technology Major Project (2025ZD18016002, China), Sichuan Science and Technology Program (2024NSFSC0048), 1.3.5 Project for Disciplines of Excellence, West China Hospital, Sichuan University (ZYGD25002), Sichuan University Postdoctoral Interdisciplinary Innovation Fund (JCXK2206).

## Declaration of competing interest

The authors declare that they have no known competing financial interests or personal relationships that could have appeared to influence the work reported in this paper.
